# Inhibitors of *Helicobacter pylori* Protease HtrA Found by ‘Virtual Ligand’ Screening Combat Bacterial Invasion of Epithelia

**DOI:** 10.1371/journal.pone.0017986

**Published:** 2011-03-31

**Authors:** Martin Löwer, Tim Geppert, Petra Schneider, Benjamin Hoy, Silja Wessler, Gisbert Schneider

**Affiliations:** 1 Institute of Organic Chemistry and Chemical Biology, Goethe-University, Frankfurt, Germany; 2 Division of Microbiology, University of Salzburg, Salzburg, Austria; 3 Department of Chemistry and Applied Biosciences, Swiss Federal Institute of Technology (ETH), Zürich, Switzerland; Charité-Universitätsmedizin Berlin, Germany

## Abstract

**Background:**

The human pathogen *Helicobacter pylori* (*H. pylori*) is a main cause for gastric inflammation and cancer. Increasing bacterial resistance against antibiotics demands for innovative strategies for therapeutic intervention.

**Methodology/Principal Findings:**

We present a method for structure-based virtual screening that is based on the comprehensive prediction of ligand binding sites on a protein model and automated construction of a ligand-receptor interaction map. Pharmacophoric features of the map are clustered and transformed in a correlation vector (‘virtual ligand’) for rapid virtual screening of compound databases. This computer-based technique was validated for 18 different targets of pharmaceutical interest in a retrospective screening experiment. Prospective screening for inhibitory agents was performed for the protease HtrA from the human pathogen *H. pylori* using a homology model of the target protein. Among 22 tested compounds six block E-cadherin cleavage by HtrA *in vitro* and result in reduced scattering and wound healing of gastric epithelial cells, thereby preventing bacterial infiltration of the epithelium.

**Conclusions/Significance:**

This study demonstrates that receptor-based virtual screening with a permissive (‘fuzzy’) pharmacophore model can help identify small bioactive agents for combating bacterial infection.

## Introduction

The Gram-negative human pathogen *Helicobacter pylori* (*H. pylori*) is a class 1 carcinogen responsible for the development of severe gastric inflammation and cancer diseases [1]. Although combination-drug therapies have been successfully applied an increasing bacterial resistance against these drugs is observed and novel intervention strategies are urgently sought for [2]. Here, we present a virtual screening technique for rapid identification of bioactive compounds together with its successful application to finding novel low molecular weight compounds against *H. pylori* infection. We recently identified the serine protease high temperature requirement A (HtrA) from *H. pylori* as a secreted virulence factor that directly cleaves the tumor suppressor E-cadherin on the surface of host cells [3]. Proteolytic cleavage of E-cadherin has been linked to the malignant progression of adenocarcinomas, rapid changes in cell adhesion, signaling, apoptosis, and contributes to an invasive mesenchymal transformation [4], [5]. The present study provides a general concept for identifying bioactive agents inhibiting HtrA-mediated E-cadherin cleavage, and therefore potentially combating bacterial pathogenesis.

It is common to distinguish between receptor-based (‘structure-based’) and ligand-based virtual screening approaches. While ligand-based virtual screening requires at least one known reference compound as a starting point, the input for structure-based virtual screening is a three-dimensional (3D) receptor model – typically an X-ray structure, or a carefully designed comparatative model of the target protein (‘homology model’) [6]–[9]. The task is to fit screening compounds into the binding site of the target, so that molecules are retrieved that are complementary to the protein cavity [10]. An early approach exploiting both shape and pharmacophoric feature complementary was LUDI [11], [12], a *de novo* design algorithm [13]. Automated ligand docking methods are widely used for receptor-based virtual screening [14], [15]. Another approach is to employ feature maps for virtual screening, *i.e.* a projection of pharmacophoric features into the binding site volume [16], and consider both ligand and structural information [17], [18]. Still, for the majority of the potential bacterial drug targets neither a reference ligand nor an experimentally determined target structure is available, thus preventing immediate application of these virtual screening methods. The increasing number of sequenced genomes, high-throughput structure determination and prediction by homology modeling [19] demand for methods that are independent from the structure of a bound reference ligand and also work on *apo*-proteins.

We here present a receptor-based virtual screening method that combines several individual strengths of the aforementioned strategies. A comparative model of the target protein is required as input, from which a predicted ligand binding site is automatically extracted and used as a shape and pharmacophoric feature template for rapid screening of large compound collections. As a result, a list of candidate compounds is suggested for *in vitro* testing. The method is based on a ‘fuzzy’ pharmacophore representation [20] of binding site features and volumes [21], [22], which tolerates inaccuracies of the target protein model. Predicted binding site features are encoded as an idealized receptor-derived ligand pharmacophore or ‘virtual ligand’ [18], so that conventional ligand-based virtual screening can be used to compare the virtual ligand with real compounds stored in databases or candidates generated by *de novo* design [13]. Here, we present the application of the virtual ligand concept to finding inhibitors of *H. pylori* protease HtrA [23].

## Results

### Model development and retrospective validation

Our virtual ligand concept uses the PocketPicker [21], [22] algorithm to calculate a discrete representation of one or more potential ligand binding pockets on the surface of a 3D protein model. For the generation of a feature map we used a subset of the LUDI rules [11], [12] to assign potential interaction points complementary to the protein residues surrounding the pocket (Table S1). The resulting three sets of discrete points for lipophilic interactions, hydrogen-bond donors, and acceptors were transferred to a continuous pharmacophore representation using LIQUID [20]. This is expected to allow for a certain degree of tolerance to account for uncertainty of protein modeling [24].

Prior to the prospective application we thoroughly scrutinized the virtual ligand approach in a retrospective virtual screening study. Full details are provided in the supporting information. Briefly, we computed the retrieval rate of known actives for a total of 18 protein targets from three different compound databases: i) the COBRA collection of drugs and lead compounds [25], ii) a collection of combinatorial Ugi-type three-component adducts [26], [27], and iii) the Maximum Unbiased Validation (MUV) set [28]. With only few exceptions, the virtual ligand method was able to retrieve a significant portion of active compounds among the top-ranking candidates, as determined by ROC analysis [29] (Table 1, Table S2, ROC-area under curve (AUC)>0.5). The full overview of the prediction performance for individual parameter combinations is presented in Tables S3, S4, S5. Compared to the overall enrichment as computed by ROC-AUC the early enrichment of known actives measured by the BEDROC score [30] was low for the majority of the examined targets, which clearly demonstrates the potential of the virtual ligand method for ‘scaffold-hooping’, *i.e.* the acceptance of different chemotypes among the top ranks of a result list. Notable improvement of prediction performance (*i.e.*, retrieval of known ligands) was achieved when the automatically predicted ligand binding cavities were manually adjusted. This resulted in an average increase of the ROC-AUC from 0.52 to 0.62 (Table 1, Table S3) and underscores the importance of correct binding site prediction and assignment for receptor-based virtual screening [31].

**Table 1:** Result averages of retrospective virtual screening.

Enzyme	Database	Pocket(s)a)	ROC-AUC ± stdev.	BEDROC ± stdev
ACE	COBRA	1	0.38±0.07	0.00±0.00
COX-2	COBRA	1	0.52±0.12	0.05±0.06
COX-2	COBRA	1b)	0.62±0.16	0.23±0.10
DHFR	COBRA	1	0.65±0.05	0.15±0.08
fXa	COBRA	1	0.52±0.14	0.04±0.03
fXa	COBRA	1, 6, 13, 17	0.74±0.09	0.14±0.07
PPARgamma	COBRA	1	0.53±0.04	0.05±0.02
Trypsin	COBRA	1	0.56±0.13	0.03±0.02
Tryptase	COBRA	2, 4, 17	0.72±0.06	0.18±0.06
UPA	COBRA	1	0.67±0.07	0.17±0.08
CathepsinG	MUV	1	0.54±0.09	0.07±0.05
Eph	MUV	2	0.52±0.03	0.05±0.02
ER-alpha	MUV	1	0.60±0.08	0.13±0.10
ER-beta	MUV	1	0.55±0.05	0.07±0.02
FAK	MUV	1	0.61±0.03	0.09±0.02
fXa	MUV	1	0.39±0.09	0.01±0.01
HIV-RT	MUV	1	0.52±0.11	0.08±0.03
Hsp90	MUV	1	0.64±0.05	0.13±0.06
PKA	MUV	1	0.51±0.08	0.05±0.02
Rho-kinase 2	MUV	1	0.50±0.07	0.04±0.02
fXa	Ugi	1, 6, 13, 17	0.58±0.03	0.15±0.02
Trypsin	Ugi	1	0.51±0.04	0.08±0.02
Tryptase	Ugi	2, 4, 17	0.65±0.06	0.51±0.11
UPA	Ugi	1	0.61±0.06	0.19±0.06

a) pockets numbered according to size based on PocketPicker (14) predictions.

b) the pocket volume was manually reduced.

ROC-AUC: Receiver-operator characteristic area under the curve; BEDROC: Boltzman-enhanced ROC (alpha = 20); ACE: angiotensin converting enzyme; COX-2: cyclooxygenase 2; DHFR: dihydrofolate reductase; fXa: factor Xa; PPARgamma: peroxisome proliferator activated receptor gamma; UPA: urokinase-type plasminogen activator; Eph: EphA4 receptor tyrosine kinase; ER-alpha: estrogen receptor alpha; ER-beta: estrogen receptor beta; FAK: focal adhesion kinase; HIV-RT: immuno-deficiency virus reverse transcriptase; Hsp90: heat-shock protein 90. See also Tables S2, S3, S4, S5.

### Prospective virtual screening

The actual prospective virtual screening study consisted of four steps: i) construction of a homology model of *H. pylori* protease HtrA, ii) identification and extraction of a ligand binding pocket of the surface of the target, iii) generation of a pharmacophoric feature map of the binding site and construction of a virtual ligand model, iv) similarity searching in a large compound collection using the virtual ligand as query.

#### Homology model

The exported protease HtrA is a serine protease and believed to play an important role in *H. pylori* induced pathogenesis [23]. It not only represents a potential target for pharmaceutical research, but inhibition by a small molecule inhibitor could be utilized to study the mechanism of *H. pylori* infection of human mucosa. We constructed a comparatative protein model derived from the protease DegP from *Escherichia coli* in its active conformation (PDB ID: 3cs0 [32], [33], 42% sequence identity to HtrA; BLAST [34] *e-*value = 7×10^−76^) as described [35].

#### Pocket extraction

**Figure 1:**
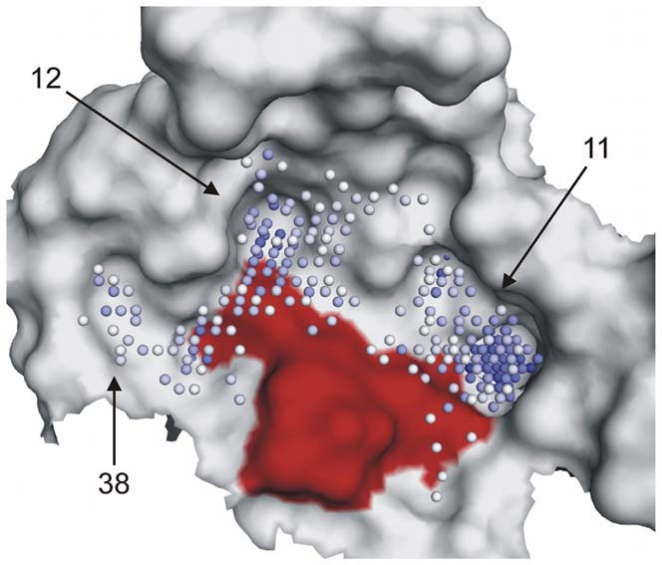
Ligand binding site predicted by PocketPicker [**21]**, [22****] on the surface of the HtrA homology model around the active site. The binding site volume is visualized by blue spheres, with darker color indicating higher buriedness. Surface patches contributed by the putative active site residues are colored in red. The numbering of the binding sites corresponds to the PocketPicker output.

We then applied virtual ligand calculation to the model starting with PocketPicker. Figure 1 presents pockets 11, 12, and 38 from the pocket prediction. DegP and *H. pylori* HtrA are known to form multimers [23], [33]. Predicted pockets larger than pocket 11 correspond to possible protein-protein interaction sites and were omitted from the present analysis. The selected pockets surround the active site residue Ser^221^ [23]. Surface loops of trypsin-like serine proteases are known to possess specificity sites [36]. These loops have similar positions in the secondary structure of serine proteases, and in the HtrA homology model actually form the selected pockets. We therefore assume that the selected pockets might represent the S1 (pocket 12), S3 (pocket 11), and S2′ (pocket 40) sites in this catalytic center of HtrA.

#### Virtual ligand model and screening

Using these pockets as input, the virtual ligand was calculated using a radius of 1.5 Å for lipophilic interaction centers, and 1.9 Å for potential hydrogen-bond donors and acceptors. Similarity between the virtual ligand and screening compounds was computed using the Manhattan distance metric. This set-up resulted from the preliminary observations made in the retrospective study for serine protease targets. In total, three virtual ligand models were built using i) all three pockets (model 1), ii) pockets 12 and 38 (model 2), iii) only pocket 11 (model 3). The models were compared against the screening database (556,763 compounds), and 26 virtual hits (Table S6) were selected from the resulting lists of 100 top-ranked compounds, ordered from the respective supplier and tested for HtrA inhibition. Manual prioritization of compounds was done to ensure that different chemotypes with different scaffolds were among the final selection; the test compounds lack apparent reactive groups, and are not too lipophilic.

### *In vitro* screening

**Figure 2:**
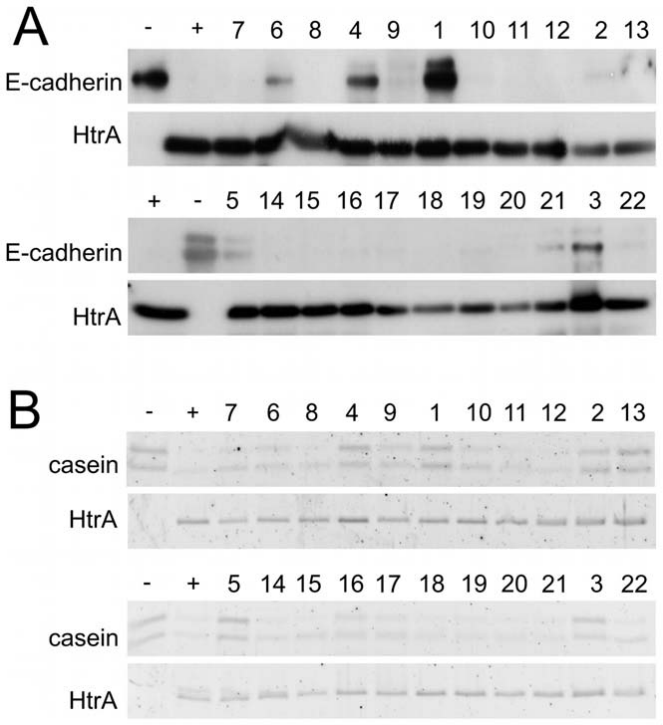
*In vitro* inhibition of HtrA-cleaved E-cadherin and casein. Incubation of E-cadherin (A) or casein (B) as substrates with HtrA led to efficient digestion in the+lane. The – lane shows the total amount of substrate that was loaded in all lanes. Screening of 22 compounds (numbers above the Western blot) was performed at a ligand concentration of 100 µM. E-cadherin was detected by Western blot, and casein was visualized by SYPRO ruby staining.

Healthy intact epithelia depend on the integrity of adhesive complexes including lateral tight junctions and E-cadherin-based adherence junctions [37]. We recently identified E-cadherin as a substrate of *H. pylori* HtrA and demonstrated that E-cadherin cleavage by HtrA results in the loss of cell-cell contact enabling the bacteria to invade the gastric epithelium [3]. We therefore tested the selected compounds for their ability to block E-cadherin cleavage by HtrA *in vitro* (Fig. 2A). From the original 26 compounds, 22 were soluble in DMSO, and six (27%) clearly inhibited proteolytic activity of HtrA (Table S6). Recombinant E-cadherin (−) was co-incubated with purified HtrA (+) and 22 test compounds. From Western blot analysis, we saw efficient inhibition of E-cadherin cleavage by HtrA by compounds **1**, **3**, and **4**, and partial inhibition by compounds **5**, **6**, and potentially **21**. The activity of compound **1** (*IC*_50_ = 26±12 µM) was reported by us previously [3] (Figure 2A). Here, we repeated the dose-response analysis corroborating this activity range (Figure S1A). At a concentration of 100 µM, both compound **1** and compound **3** efficiently blocked E-Cadherin *in vitro* (Figure S1A, Figure S1B). Notably, titration of compound **3** revealed only a slightly different inhibitory activity of E-cadherin cleavage by HtrA (Figure S1B). We additionally used casein as an artificial substrate for HtrA [32] leading to similar results (Figure 2B). Slight differences of HtrA digestion of E-cadherin in comparison to casein are visible in Figure 2B, which might be caused by differences in substrate recognition. In particular, compound **21** has a weak inhibitory effect on E-cadherin cleavage but not on casein cleavage. We therefore did not consider compound **21** for further analysis. It is reasonable to assume that HtrA possesses a substrate specificity pocket that tolerates several residue patterns in the substrate sequence. We are currently testing this hypothesis. For the most potent inhibitor **1**, we determined a purity of 92% (Figure S2) and performed an additional direct inhibition assay using fluorescence-labeled casein as substrate. Casein cleavage was reduced by approximately 27% in the presence of the inhibitor (Figure S3).

The outcome of this study confirms that the virtual ligand concept may be used for hit retrieval, even in combination with a homology model of the protein target. It might thus be regarded as a complement to automated ligand docking and re-scoring, and related receptor-derived pharmacophore concepts [38]–[41]. Docking of all 26 compounds into the area defined by the virtual ligand models supports this assumption, as there is no apparent correlation between the docking score value and the actual inhibitory activity of the compounds (Table S6).

### Structure-activity relationship

**Figure 3:**
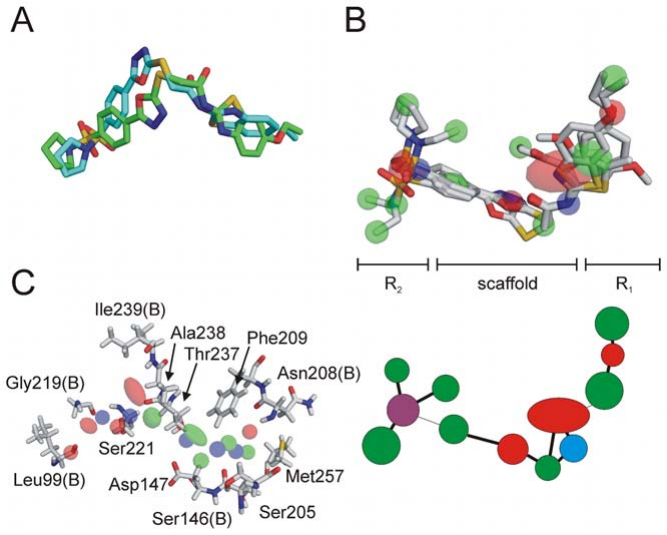
Consensus LIQUID pharamacophore model and binding site of HtrA. (**A**) Superimposition of the docked (cyan) and the database (green) conformation of compound **1**. (**B**) Flexible alignment and LIQUID consensus pharmacophore model of inhibitor compounds **1**, **4**, **5** and **6**. Below, a 2D graph representation of the model is shown. Red spheres indicate a hydrogen-bond acceptor, blue a hydrogen-bond donor, and green a lipophilic group. The purple sphere indicates an acceptor and/or a donor. (**C**) LIQUID consensus pharmacophore model of compounds **1**, **2**, **3**, **4**, **5** and **6**, placed in the binding site of HtrA. Residues possibly interacting with the pharmacophore features are shown and labeled. If only a backbone interaction is possible, a ‘B’ was added to the residue number. Note that only features are shown that are in vicinity to protein residues.

Active compounds **1**, **2**, **4**–**6** were identified by virtual ligand model 1, and active compound **3** was found with model 3. Apparently, model 2 was unsuitable for hit retrieval. This model did not include pocket 11 indicating that this sub-pocket might be important for substrate recognition (Figure 1). Compounds **1**, **2**, **4**–**6** share a common scaffold (Figure S4A) decorated by two side chains (R_1_ and R_2_ in Figure 3A,B). Figure S4B presents the best scoring docking pose obtained for compound **1** (favorable GOLD *ASPscore* = 18), and Figure S4C presents superimposed docked conformations of all inhibitory compounds. Overall, a similar common bound conformation can be assumed. According to the docking poses obtained, the ring system of the R_2_ group of compound **1** interacts with Phe^209^, and the terminal methyl is placed in lipophilic pocket 11, where the interaction is mediated by the side-chains of Ile^253^ and Met^257^. The same interaction points were predicted for compound **5** but not for compounds **4** and **6**, which have bulkier R_1_ substituents. As these do not fit into pocket 11, their docking poses with a flipped scaffold received higher scores. In the flipped orientation the bulky R_1_ substituents are located near pocket 38, which is wider than pocket 11, and the oxadiazole nitrogen atoms do not form hydrogen-bonds to the backbone of Ile^239^, in contrast to compound **1**. This could explain the lower activity of compounds **4** and **6**. In our binding model, the R_2_ groups of compounds **1** and **5** are placed in pocket 38, which allows an oxygen atom of the sulfone group of compound **1** to form a hydrogen bond with the backbone nitrogen of Gly^219^. The corresponding sulfonyl oxygen of compound **5** cannot be placed in this favorable position. The pyrrolidine side-chain of compound **1** may also interact with the hydrophobic environment of pocket 38. Summarizing, these observations from the predicted docking modes could explain the lesser activity of compound **5** compared to compound **1**.

Although compounds **7**, **11** and **13** share the scaffold shown in Figure 3A, they do not exhibit inhibitory activity towards HtrA. Compound **7** possesses the bulkiest R_1_ group of this series, which might explain its inactivity. Compounds **11** and **13** are strikingly similar to inhibitory compound **4**. Compound **11** only differs by a 3,4-configuration of the dimethoxybenzene group instead of a 3,5-configuration. Such a small change of structure resulting in a complete activity loss suggests a steep structure-activity landscape [42]. Compound **13** also has a substituent in the *para*-position of the R_1_ benzene suggesting this substituent might not be favorable. Assuming that compounds **11** and **13** adopt a similar scaffold orientation as compound **4**, the *para*-substituents of **11** and **13** would point into a region outside the predicted pocket, without any protein atoms as interaction partners (Figure S4D). A possible explanation is that compound **4** actually adopts a different preferred binding mode, which was not detected in the docking simulations.

We superimposed docked conformations of compound **1** with those found in the virtual screening study by rigid body alignment (MOE version 2007.09). Both conformations feature a similar bend (Figure 3A). This indicates that the virtual ligand algorithm successfully encoded shape information about the binding site. Due to the fact that the results – and consequently our interpretations – of the docking procedure might be erroneous we performed an additional flexible alignment of compounds **1**, **4**, **5**, and **6**, and calculated a consensus pharmacophore model (Figure 3B). This model can serve as a starting point for further virtual screenings based on ligand information alone. Note that this model partly differs from the docking results, as the orientation of the scaffold is flipped for compounds **4** and **6**. Therefore, we cannot unambiguously suggest a consensus binding pose for all inhibitors.

For identification of protein residues possibly interacting with the bound inhibitors a hybrid approach was used including both ligand and binding site information. Docked conformations of all inhibitors were superimposed and a pharmacophore model was calculated with LIQUID. This model was placed in the binding site and visually investigated for potential ligand-receptor interactions. Figure 3C presents this model and the corresponding residues, which may serve as a guideline for HtrA mutation studies to determine the actual pharmacophoric interaction pattern.

### Cell-based studies

**Figure 4:**
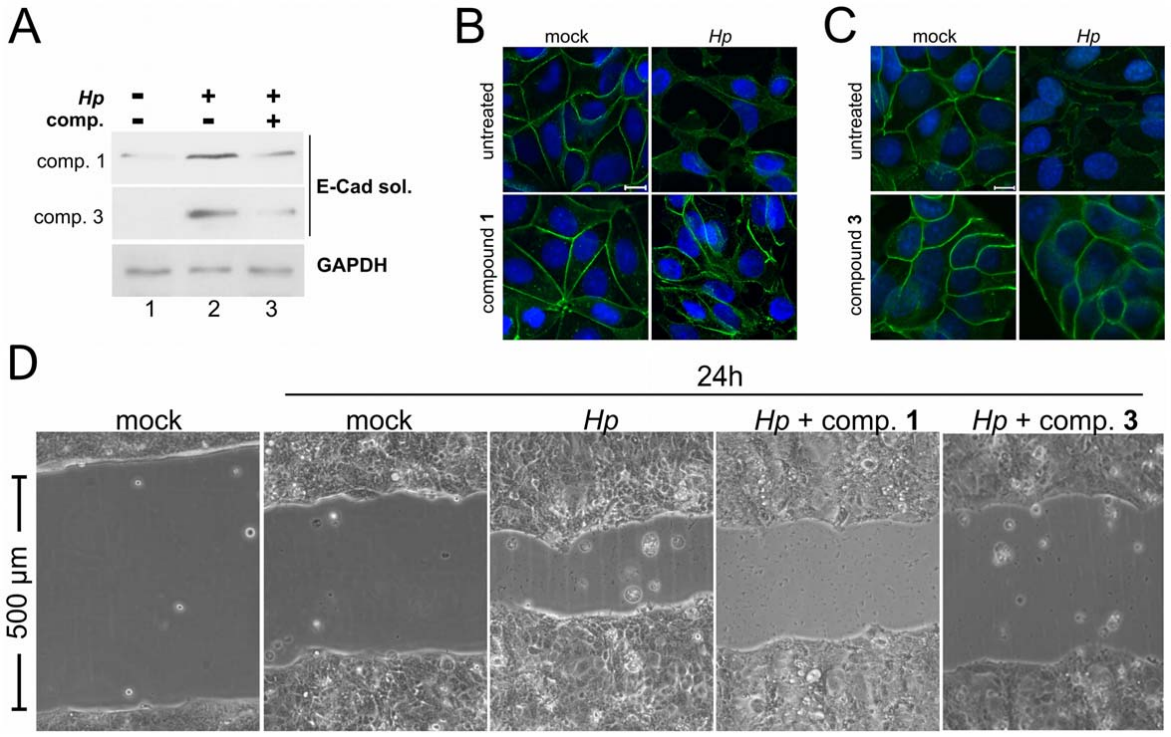
Effects of compounds 1 and 3 on E-cadherin-mediated cell adhesion. (A) MKN-28 cells were infected with *H. pylori* for 16 h. Where indicated, cells were co-treated with 100 mM compound 1 or compound 3. The formation of soluble E-cadherin fragment in the supernatant of cells was detected by Western blot using an antibody detecting the extracellular E-cadherin domain. Equal amounts of cells were demonstrated by the detection of GAPDH in protein lysates. (B) Confluent MCF-7 cells were untreated (mock) or infected with *H. pylori* for 16 h (right), which resulted in a loss of E-cadherin-mediated cells adhesion and a scattered phenotype. Cells were co-treated with a 100 µM solution of compound **1** (**B**) or **3** (**C**), thereby preventing dissociation of E-cadherin-mediated cell contacts and the scattered phenotype. E-cadherin (green) was stained using an antibody detecting the intracellular domain. Nuclei (blue) were stained using DAPI. Scale bar: 10 µm. (**D**) Compounds **1** and **3** delay wound healing of *H. pylori*-infected MKN-28 cells. MKN-28 cells were seeded on cell culture dishes equipped with a silicone insert, which was removed when cells reached confluence. The obtained scratch of exactly 500 µm was monitored for 24 hours while cells were treated with *H. pylori* and compound **1** or **3**.

To probe whether compounds **1** and **3** – as representatives of the two prevalent scaffolds among the top-ranking hits – are able to prevent disruption of epithelia by *H. pylori*, we investigated their effect on functional adhesion of epithelial cells. Confluent MCF-7 and MNK-28 cells develop functional E-cadherin-dependent intercellular adhesions, which are actively disrupted by *H. pylori* after HtrA-induced shedding of the ectodomain of E-cadherin [3], [43]. We tested if compounds **1** and **3** might be suitable to inhibit HtrA-triggered E-cadherin cleavage in *H. pylori* infections (Figure 4). Cells were either colonized with *H. pylori* alone (Figure 4A, lane 2), in combination with 100 µM compound **1** or compound **3** (Figure 4A, lane 3), or left uninfected and untreated by any of the two compounds (Figure 4A, lane 1). E-cadherin cleavage was analyzed by the detection of soluble E-cadherin in the supernatants of cells (‘E-cad sol.’). Both compounds decreased the formation of soluble E-cadherin fragments upon infection with *H. pylori* supporting these compounds as functional small molecule inhibitors of HtrA. Performing confocal laser scanning microscopy, we detected E-cadherin in the plasma membrane of uninfected MCF-7 cells (Figure 4B and 4C, ‘mock’). After colonization with *H. pylori* membrane localization of E-cadherin was strongly relieved and intercellular adhesions were disrupted (Figure 4B and 4C, ‘*Hp*’). Compounds were added to MCF-7 cells prior to *H. pylori* infection and did not affect E-cadherin staining or cell morphology. Finally, both compounds **1** and **3** efficiently blocked *H. pylori*-induced loss of intercellular adhesions and E-cadherin staining, and judging from cell morphology compound **3** appears to be the more effective agent (Figure 4B and 4C, lower right panel).

Ectodomain shedding of E-cadherin promotes cell proliferation, migration, and invasion and is considered a relevant and important cancer biomarker [3]. To investigate biological significant inhibition of HtrA-mediated E-cadherin cleavage, we performed a wound-healing assay as a model of cellular proliferation and migration. A confluent cell monolayer exhibiting a 500 µm thick ‘scratch’ was left untreated, infected with *H. pylori*, or treated with compound **1** or **3** together with *H. pylori* for a period of 24 hours. Direct comparison of MKN-28 cells revealed that inhibition of HtrA by compounds **1** and **3** led to an obvious delay of wound closure (Figure 4D). Although we cannot exclude the possibility that these compounds might also interfere with proliferation- or migration-associated signal transduction pathways, these data imply that the successful pharmacological inhibition of HtrA-mediated E-cadherin cleavage has a notable influence on cellular proliferation and migration.

## Discussion

In this work we present the successful application of virtual screening based on the automated extraction of a ligand-binding site and receptor-based pharmacophores. ‘Virtual ligand’ screening for inhibitors of *H. pylori*-secreted HtrA resulted in the identification of several hits. Compounds **1** and **3** exhibit pronounced bioactivity in *in vitro* infection experiments. These results confirm the applicability of homology model-based virtual screening to hit finding. In this preliminary study, several scaffold structures were retrieved from a large screening compound collection, which offer rich opportunity for hit profiling and eventual hit-to-lead optimization. Retrospective screening experiments showed that the definition of the binding site volume critically affects screening performance, and final manual control and selection of (sub-)pockets appears to be mandatory for the retrieval of bioactive compounds. The prospective screening experiment demonstrates that identification of various bioactive chemotypes is possible, and a preliminary structure-activity relationship may be deduced from these data. Certainly, the overall performance of the virtual ligand concept will remain target-dependent. The best inhibitor **1** exhibits sustained bioactivity *in vitro* and effectively prevents the disruption of epithelial cells by *H. pylori*. We wish to stress that this substance should be considered as a ‘tool compound’ rather than a pharmaceutical lead structure. Its potency is moderate and we identified potential aqueous solubility issues. Compound **3** appears to be even more effective in cell culture (Figure 4) and possesses a promising alternative scaffold for actual lead compound development. With a total of six inhibitors available, additional virtual screening runs and *de novo* design methods can now be applied for HtrA inhibitor optimization. These first-in-class HtrA inhibitors will help to gain new insights into the relationships between human host cells and *H. pylori* on the molecular level.

## Materials and Methods

### Virtual ligand modeling

The virtual ligand was calculated in four steps:

- The protonation state of the target structure was determined with MOE Protonate3D (MOE version 2007.09 The Molecular Operating Environment, Chemical Computing Group Inc., Montreal, Canada).
- Potential ligand binding sites were predicted by PocketPicker [21], [22]. In brief, PocketPicker uses a geometric approach to identify those nodes of a grid (1 Å spacing placed around the protein), which are buried in clefts of the protein surface. These nodes are clustered to disjunct sets using a calculated buriedness value. Each set of nodes is assumed to represent the volume and the shape of a potential ligand binding site.
- One or more pocket models calculated in the previous step were used as the input for the further processing. The set of residues including a non-hydrogen atom with a minimal distance to one of the nodes of the respective model was calculated. This set is assumed to be the set of interacting pocket residues. The program iterates over all atoms of the set and all nodes of the pocket model and checks for each node/atom pair if one of the rules given in Table S1 is satisfied. For rules 1 and 2 this was done by calculating the distance d of the optimal position of an interaction partner of the atom and the pocket node under observation (Eq. 1).
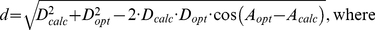
(1)
*D_calc_* and *A_calc_* are the calculated distance and angle values between the points required by the respective rule and *D_opt_* and *A_opt_* the optimal values given by the rule. The value of *d* should be zero; since the distribution of the pocket nodes is discrete a tolerance of 0.9 Å was allowed. This value is close to half the maximal distance of two nodes, which is given by (3^1/2^)/2 for the PocketPicker grid, and ensures that at least one node satisfies the rule if the interaction points into the space defined by the pocket model. For rule 3 and 4, the Euclidian distance between the points under investigation was compared to the optimum value (tolerance: 0.5 Å). The coordinates of the corresponding pocket nodes satisfying a rule were stored in separate sets for each interaction type.
  The given rules were taken from the *de novo* design program LUDI [11], [12] and represent a subset of the original LUDI rules. Aromatic carbon atoms were treated as aliphatic/lipophilic.
- The program LIQUID [20] was used for clustering the nodes in the sets of each interaction type. A local feature density (LFD) was used to determine if a node belongs to a cluster. Using principal component analysis, LIQUID calculates a trivariate Gaussian distribution (*trivG*) [20] for each cluster that represents so-called ‘fuzzy’ potential pharmacophore points (fPPP). The set of the fPPPs for all interaction types was used to calculate a 120-dimensional correlation vector, the ‘virtual ligand’ (Eq. 2).
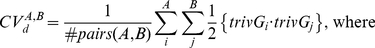
(2)
*A* and *B* are interaction types under investigation; *d* is one of twenty distance intervals with a width of 1 Å (from 0 to 20 Å); *i* and *j* are fPPPs of types *A* or *B*, respectively.

The whole algorithm was implemented in the programming language Java [44] using the Chemistry Development Kit (CDK) [45].

### Data sets and data set preparation

For the retrospective virtual screening experiments we used the COBRA dataset (version 6.1) of bioactive compounds [25], a compilation of 15,540 three-component Ugi reaction products [18], [26], [27], and the Maximum Unbiased Validation (MUV) sets [28]. The Ugi products had been tested for inhibition of five serine proteases: chymotrypsin, factor Xa, trypsin, tryptase, and urokinase-type plasminogen activator. Only a subset of the targets included in the COBRA database was selected for the screening experiments, and some of the MUV datasets had to be excluded due to unavailability of protein models in the protein database (PDB) [32]. For prospective screening, the compound collections (Gold and Platinum, 04.2007) from Asinex Ltd. (Moscow, Russia) and Specs v04.2007 (Delft, The Netherlands) were pooled and served as screening database. MOE conformation import (MOE version 2007.09) was used to calculate up to 250 conformers for each molecule in the screening database. LIQUID was used to derive the pharmacophore model and correlation vector for each conformer.

### Virtual screening parameters

LIQUID employs several parameters for the calculation of pharmacophore models: cluster radius for hydrogen-bond acceptor, donor and lipophilic clusters and scaling of correlation vectors (no scaling, block scaling to range [0,1], and vector scaling to range [0,1]). The cluster radii were set to the default value of 1.9 Å, while all scaling options were tested. Also, for distance calculation both Manhattan and Euclidian distance and the cosine similarity were used. Testing was done by ten-times leave-group-out cross-validation with random 50+50 splits [46]. For performance evaluation we used the receiver operating characteristic area under curve (ROC-AUC) [29] and the Boltzmann-enhanced discrimination of receiver operating characteristic (BEDROC, with alpha = 20) [30]. Ligand docking was done with the software GOLD and the ASP scoring function [47].

### Homology modeling

A homology model of the protease HtrA of *Helicobacter pylori* was built using MOE Homology (MOE version 2007.09) and the structure model of the protease HtrA of *Escherichia coli* as template (PDB ID 3cs0), as described [35].

### Experimental procedures

Cloning, expression and purification of HtrA of *H. pylori* was performed as described previously [23]. The ordered test compounds were dissolved in DMSO and diluted to stock concentration. 0.5 µg HtrA was incubated with the corresponding amount of the respective compound and 0.1 µg E-cadherin/Fc-Chimera (R&D Systems) or casein in 50 mM 4-(2-hydroxyethyl)-1-piperazineethane sulfonic acid (HEPES) buffer (pH 7.4) for two hours at 37°C. The reaction was stopped by boiling for five minutes and analyzed by SDS-PAGE and SYPRO Ruby staining (Invitrogen) or Western-blotting and immunostaining with anti-E-cadherin antibody (Santa Cruz Biotechnology). A film was exposed to the ECL/HRP chemo-luminescence reaction and scanned, or data were acquired directly by a FUSION-FX7 camera (Vilber Lourmat). Background noise filtering by a rolling-ball algorithm and the measurement of brightness densities was performed using ImageJ (version1.41o) [48].

### Cell culture, bacteria and infection experiments

Human breast cancer cells (MCF-7, LGC Standards GmbH, Germany, http://www.lgcstandards-atcc.org) and human gastric cancer cells (MKN-28 [3]) were grown in DMEM medium (Biochrom, Germany) and 10% FCS (Biowest, France) in a humidified 10% CO2 atmosphere at 37°C. Cells were seeded on glass slides 48 hours before infection. 1–2 h prior to infection medium was replaced by serum-free DMEM. *H. pylori* strain Hp26695 was cultured on agar plates containing 10% horse serum under micro-aerophilic conditions at 37°C for 48 hours. For infection, bacteria were harvested in PBS Dulbecco's medium, pH 7.4, added to the host cells at a multiplicity of infection (MOI) of 100 for 16 h. Cells were fixed in 4% paraformaldehyde in PBS, and permeabilized in 0.2% Triton X-100 in PBS. Immunostaining was performed using anti-E-cadherin (cl. 36 detects the intracellular domain, BD Biosciences), For nuclei staining, 4′,6-diamin-2-phenylin-dol-dihydrochloride (DAPI, Roche) was used according to the manufacturer's instructions. Samples were analyzed by confocal laser scanning microscopy using a Zeiss LSM 510 Meta confocal microscope. Images were processed using Corel Photopaint (Corel Inc., Ottawa, Canada). Supernatants of cells were analyzed for E-cadherin cleavage by the detection of the soluble E-cadherin fragment by Western blot analysis as described above. Cells were then lysed in 20 mM Tris (pH 7.5), 0.42 M NaCl, 1.5 mM MgCl_2_, 0.2 mM EDTA, 10 mM K_2_HPO_4_, 1 mM Na_3_VO_4_, 10 mM NaF, 1.25% Nonidet P-40 and 10% glycerol. Aliquots were analyzed for GAPDH expression using an anti-GAPDH antibody (Abcam) to demonstrate equal numbers of cells. For the wound healing assay a silicone insert was placed on a cell culture surface before seeding gastric epithelial MKN-28 cells. When cells reached confluence, the silicone insert was removed resulting in 500 µm thick ‘scratch’. The cells were either left untreated, infected with *H. pylori*, or treated with test compounds together with *H. pylori* for 24 h and monitored by an inverse microscope.

## Supporting Information

Figure S1 ***In vitro*** **inhibition of E-cadherin cleavage by HtrA in the presence of different concentrations of compound 1 (1, 3, 10, 30 100 µM) (A) and compound 3 (5, 10, 50, 75, 100 µM) (B). E-cadherin and HtrA were detected by Western blot.**
(PDF)

Figure S2 **Purity analysis of compound 1.** We performed HPLC and mass detection of compound **1** in 100% DMSO. Compound purity was determined to be 92%, and the correct mass peak was detected at 546 Da. (**A**) HPLC report for compound **1** (Shimadzu LCMS2020). (**B**) Mass spectrum recorded for compound **1** (Shimadzu LCMS2020).
(PDF)

Figure S3 **Enzyme inhibition assay.** Raw data (triplicates) obtained for compound **1** (termed “HHI” in this plate reader protocol) in the protease inhibition assay. Inhibition of HtrA by compound **1** was tested in a fluorimetric protease assay as described (Protease Detection Kit, Jena Bioscience, Germany; substrate: casein). 50 µl incubation buffer were mixed with 100 µl sample and 50 µl casein stock solution as specified by the vendor, and incubated for 3 h at 37°C. 500 µl precipitation reagent were added and incubated for 30 min at 37°C. The reaction vials were centrifuged at 12.000 g for 5 min. 400 µl of the supernatant were mixed with 600 µl assay buffer. Fluorescence was measured in a Tecan M1000 spectrometer (excitation wavelength: 490 nm, emission wavelength: 525 nm), in a Greiner 384 well plate (flat bottom black plate) holding 100 µl per well. Final concentration of HtrA: ca. 10 nM, compound **1**: 170 µM.
(PDF)

Figure S4 **(A) Scaffold of compounds 1, 4, 5 and 6 (inhibitory activity), and 7, 11, 13 (no inhibitory activity).** (**B**) Superposition of docking poses of compounds **1** (cyan), **4** (pink), **5** (blue) and **6** (magenta). (**C**) Same as (**B**) including compounds **2** (grey) and **3** (orange). (**D**) Superposition of compounds **7**, **11**, **13**.
(PDF)

Table S1 **Idealized geometric interaction rules used for the calculation of the virtual ligand model (8).**
(DOC)

Table S2 **Results of retrospective screening; averaged over all targets.**
(DOCX)

Table S3 **Results of retrospective screening using the COBRA database.** Three different dissimilarity metrics were used a) Euclidian distance, b) Manhattan distance c) Cosine similarity. The highest ROC-AUC for each model is marked in bold.
(DOCX)

Table S4 **Results of retrospective screening using the UGI database.** Three different dissimilarity metrics were used a) Euclidian distance, b) Manhattan distance c) Cosine similarity. The highest ROC-AUC for each model is marked in bold.
(DOCX)

Table S5 **Results of retrospective screening using the MUV database.** Three different dissimilarity metrics were used a) Euclidian distance, b) Manhattan distance c) Cosine similarity. The highest ROC-AUC for each model is marked in bold.
(DOCX)

Table S6 **Structures and activities (inhibition of HtrA) of the inhibitory compounds, ordered according to falling inhibitory activity.** The Gold docking rank calculated for all 26 ordered compounds as well as the Gold ASP score in brackets is shown in column 5.
(DOCX)
