# Supplementary figures and images for: Inhibitors of Helicobacter pylori Protease HtrA Found by ‘Virtual Ligand’ Screening Combat Bacterial Invasion of Epithelia
Source: PLoS One. 2011 Mar 31;6(3):e17986. doi: 10.1371/journal.pone.0017986 (PMC3069028; doi:10.1371/journal.pone.0017986)

**A**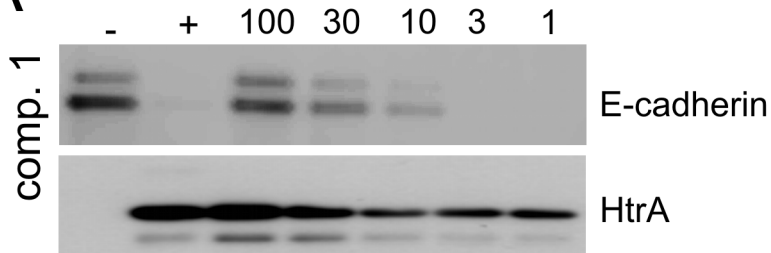**B**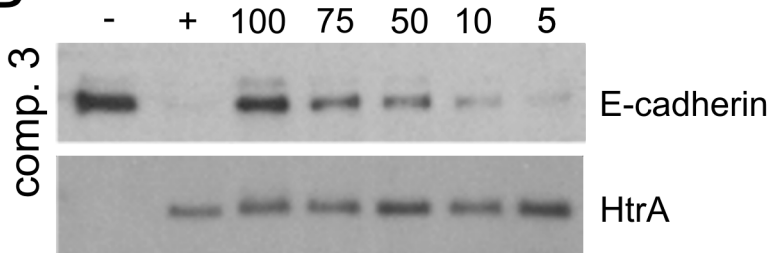

Supplement: Figure S1 — In vitro inhibition of E-cadherin cleavage by HtrA in the presence of different concentrations of compound 1 (1, 3, 10, 30 100 µM) (A) and compound 3 (5, 10, 50, 75, 100 µM) (B). E-cadherin and HtrA were detected by Western blot. (PDF) [file pone.0017986.s001.pdf]

A

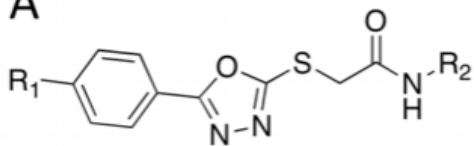

B

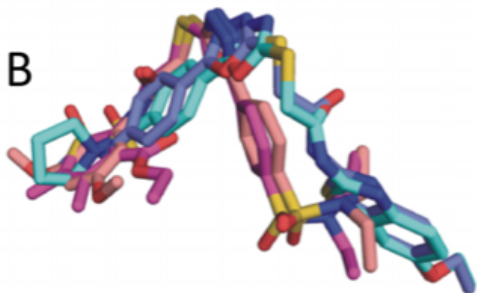

C

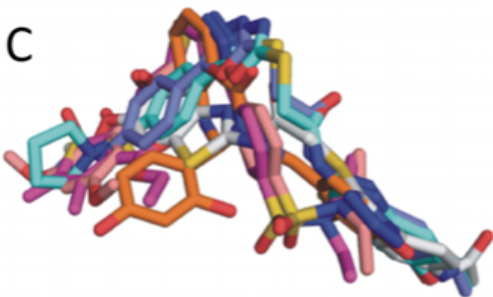

D

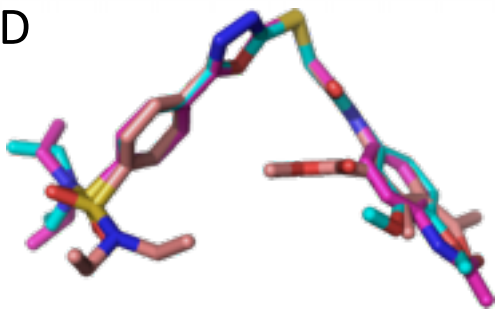

Supplement: Figure S4 — (A) Scaffold of compounds 1, 4, 5 and 6 (inhibitory activity), and 7, 11, 13 (no inhibitory activity). (B) Superposition of docking poses of compounds 1 (cyan), 4 (pink), 5 (blue) and 6 (magenta). (C) Same as (B) including compounds 2 (grey) and 3 (orange). (D) Superposition of compounds 7, 11, 13. (PDF) [file pone.0017986.s004.pdf]
