# Supplementary material for: Inhibitors of Helicobacter pylori Protease HtrA Found by ‘Virtual Ligand’ Screening Combat Bacterial Invasion of Epithelia
Source: PLoS One. 2011 Mar 31;6(3):e17986. doi: 10.1371/journal.pone.0017986 (PMC3069028; doi:10.1371/journal.pone.0017986)

A

C:\LabSolutions\Data\Project1\HtrAI8HPLC1.lcd

Acquired by	: System Administrator
Sample Name	: HtrAI8HPLC
Sample ID	:
Tray#	: 1
Vial#	: 56
Injection Volume	: 50
Data File	: C:\LabSolutions\Data\Project1\HtrAI8HPLC1.lcd
Method File	: C:\LabSolutions\Data\Project1\HZ142.lcm
Report Format File	: C:\LabSolutions\System\DEFAULT.lsr
Month-Day Acquired	: 8/19/2010
Month-Day Processed	: 8/19/2010

<Chromatogram>

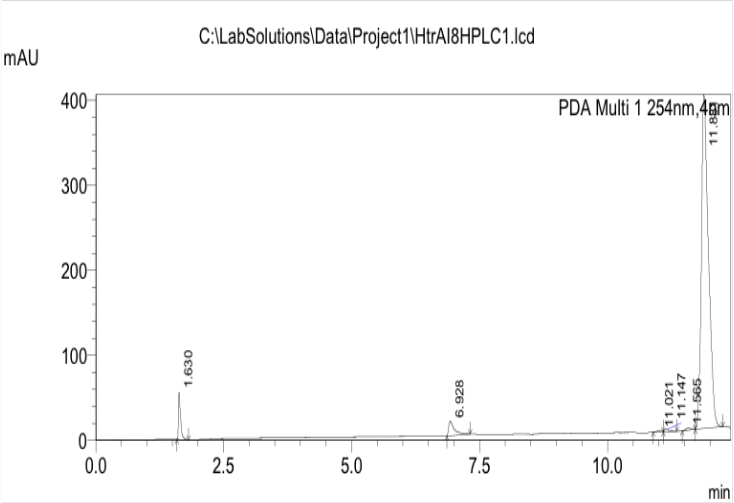

Peak Table

PDA Ch1 254nm

Peak#	Ret. Time	Area%	Area	Height	Name
1	1.630	3.843	157754	55183	RT:1.630
2	6.928	3.242	133085	16789	RT:6.928
3	11.021	0.296	12140	1516	RT:11.021
4	11.147	0.479	19661	2303	RT:11.147
5	11.565	0.437	17942	2224	RT:11.565
6	11.881	91.704	3764844	391185	RT:11.881
Total		100.000	4105426	469200	

B

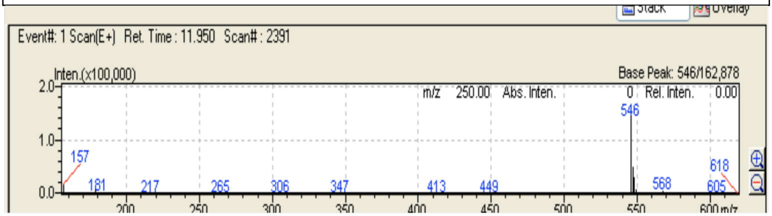

Supplement: Figure S2 — Purity analysis of compound 1. We performed HPLC and mass detection of compound 1 in 100% DMSO. Compound purity was determined to be 92%, and the correct mass peak was detected at 546 Da. (A) HPLC report for compound 1 (Shimadzu LCMS2020). (B) Mass spectrum recorded for compound 1 (Shimadzu LCMS2020). (PDF) [file pone.0017986.s002.pdf]
