# Supplementary material for: Inhibitors of Helicobacter pylori Protease HtrA Found by ‘Virtual Ligand’ Screening Combat Bacterial Invasion of Epithelia
Source: PLoS One. 2011 Mar 31;6(3):e17986. doi: 10.1371/journal.pone.0017986 (PMC3069028; doi:10.1371/journal.pone.0017986)
Supplement: Figure S3 — Enzyme inhibition assay. Raw data (triplicates) obtained for compound 1 (termed “HHI” in this plate reader protocol) in the protease inhibition assay. Inhibition of HtrA by compound 1 was tested in a fluorimetric protease assay as described (Protease Detection Kit, Jena Bioscience, Germany; substrate: casein). 50 µl incubation buffer were mixed with 100 µl sample and 50 µl casein stock solution as specified by the vendor, and incubated for 3 h at 37°C. 500 µl precipitation reagent were added and incubated for 30 min at 37°C. The reaction vials were centrifuged at 12.000 g for 5 min. 400 µl of the supernatant were mixed with 600 µl assay buffer. Fluorescence was measured in a Tecan M1000 spectrometer (excitation wavelength: 490 nm, emission wavelength: 525 nm), in a Greiner 384 well plate (flat bottom black plate) holding 100 µl per well. Final concentration of HtrA: ca. 10 nM, compound 1: 170 µM. (PDF) [file pone.0017986.s003.pdf]

| | | | | | | |
|---|---|---|---|---|---|---|
| Plate | | | Greiner 384 Flat Bottom Black Polystyrol [GRE | | | |
| Plate-ID (Stacker) | | | | | | |
| | | | | | | |
| | | | | | | |
| Label: Label2 | | | | | | |
| Mode | | | Fluorescence Top Reading | | | |
| Excitation Wavelength | | | 490 | nm | | |
| Emission Wavelength | | | 525 | nm | | |
| Excitation Bandwidth | | | 5 | nm | | |
| Emission Bandwidth | | | 5 | nm | | |
| Gain | | | 104 | Optimal (100%) | | |
| Number of Flashes | | | 50 | | | |
| Flash Frequency | | | 400 | Hz | | |
| Integration Time | | | 20 | µs | | |
| Lag Time | | | 0 | µs | | |
| Settle Time | | | 0 | ms | | |
| Z-Position (Calculated From: K1) | | | 23542 | µm | | |
| Part of Plate | | | K1-N12 | | | |
| Start Time: | 24.08.2010 15:43:49 | | | | | |
| | | | | | | |
| | Temperature: 26.6 °C | | | | | |
| | | | | | | |
| | 8233 | 8420 | 7905 | 6786 | 5934 | 5119 |
| HtrA | + | + | + | + | + | + |
| HHI | - | - | - | + | + | + |
| | | | | | | |
| End Time: | 24.08.2010 15:44:06 | | | | | |
