# Supplementary material for: Inhibitors of Helicobacter pylori Protease HtrA Found by ‘Virtual Ligand’ Screening Combat Bacterial Invasion of Epithelia
Source: PLoS One. 2011 Mar 31;6(3):e17986. doi: 10.1371/journal.pone.0017986 (PMC3069028; doi:10.1371/journal.pone.0017986)
Supplement: Table S1 — Idealized geometric interaction rules used for the calculation of the virtual ligand model (8). (DOC) [file pone.0017986.s005.doc]

**Table S1.** Idealized geometric interaction rules used for the calculation of the virtual ligand model (8).

| Rule | Protein atom | Interaction type | Criteria |
| --- | --- | --- | --- |
| 1 | H-donor (NH, OH) | H-acceptor (A) | *distance*_H…A_ = 1.9 Å  *angle*_N/O-H…A_ = 180° |
| 2 | Oxygen (C=O, R_1_-C-R_2_) | H-donor (D) | *distance*_O…D_ = 1.9 Å  *angle*_C-O…D_ = 120° |
| 3 | Nitrogen (unprotonated) | H-donor (D) | *distance*_N…D_ = 1.9 Å |
| 4 | Aliphatic carbon | Lipophilic (L) | *distance*_C…L_ = 4 Å |
