# Supplementary material for: Inhibitors of Helicobacter pylori Protease HtrA Found by ‘Virtual Ligand’ Screening Combat Bacterial Invasion of Epithelia
Source: PLoS One. 2011 Mar 31;6(3):e17986. doi: 10.1371/journal.pone.0017986 (PMC3069028; doi:10.1371/journal.pone.0017986)
Supplement: Table S2 — Results of retrospective screening; averaged over all targets. (DOCX) [file pone.0017986.s006.docx]

**Table S2.** Results of retrospective screening; averaged over all targets.

|  |  |  | no scaling | | | | block scaling | | | | scaling to one | | | |
| --- | --- | --- | --- | --- | --- | --- | --- | --- | --- | --- | --- | --- | --- | --- |
| database | metric | cluster radius [Å] | ROC-AUC | (σ) | BEDROC | (σ) | ROC-AUC | (σ) | BEDROC | (σ) | ROC-AUC | (σ) | BEDROC | (σ) |
| COBRA | E | 1.5 | 0.60 | (0.13) | 0.09 | (0.05) | 0.55 | (0.11) | 0.05 | (0.03) | 0.59 | (0.19) | 0.11 | (0.09) |
|  |  | 2 | 0.61 | (0.11) | 0.11 | (0.08) | 0.58 | (0.11) | 0.08 | (0.08) | 0.60 | (0.19) | 0.14 | (0.13) |
|  |  | 4 | 0.58 | (0.12) | 0.10 | (0.07) | 0.59 | (0.09) | 0.08 | (0.09) | 0.57 | (0.19) | 0.13 | (0.13) |
|  | M | 1.5 | 0.60 | (0.14) | 0.08 | (0.06) | 0.57 | (0.13) | 0.06 | (0.06) | 0.58 | (0.21) | 0.10 | (0.09) |
|  |  | 2 | 0.62 | (0.14) | 0.12 | (0.10) | **0.63** | (0.11) | **0.11** | (0.11) | 0.59 | (0.18) | 0.13 | (0.13) |
|  |  | 4 | 0.58 | (0.18) | 0.10 | (0.11) | 0.62 | (0.14) | 0.10 | (0.12) | 0.55 | (0.21) | 0.12 | (0.15) |
|  | C | 1.5 | 0.59 | (0.14) | 0.10 | (0.08) | 0.52 | (0.10) | 0.04 | (0.02) | 0.59 | (0.14) | 0.10 | (0.08) |
|  |  | 2 | 0.62 | (0.14) | 0.14 | (0.12) | 0.57 | (0.12) | 0.07 | (0.08) | 0.62 | (0.14) | 0.14 | (0.12) |
|  |  | 4 | 0.61 | (0.14) | 0.13 | (0.10) | 0.57 | (0.14) | 0.08 | (0.09) | 0.61 | (0.14) | 0.13 | (0.11) |
| MUV | E | 1.5 | **0.61** | (0.08) | **0.11** | (0.07) | 0.49 | (0.11) | 0.04 | (0.03) | 0.53 | (0.09) | 0.08 | (0.06) |
|  |  | 2 | 0.56 | (0.09) | 0.11 | (0.09) | 0.51 | (0.13) | 0.07 | (0.07) | 0.52 | (0.12) | 0.07 | (0.06) |
|  |  | 4 | 0.59 | (0.10) | 0.11 | (0.09) | 0.55 | (0.10) | 0.07 | (0.07) | 0.53 | (0.12) | 0.08 | (0.06) |
|  | M | 1.5 | 0.61 | (0.07) | 0.09 | (0.03) | 0.49 | (0.11) | 0.05 | (0.03) | 0.55 | (0.09) | 0.07 | (0.04) |
|  |  | 2 | 0.54 | (0.08) | 0.10 | (0.09) | 0.52 | (0.12) | 0.06 | (0.03) | 0.51 | (0.09) | 0.06 | (0.04) |
|  |  | 4 | 0.60 | (0.11) | 0.10 | (0.10) | 0.55 | (0.13) | 0.08 | (0.07) | 0.53 | (0.11) | 0.07 | (0.05) |
|  | C | 1.5 | 0.53 | (0.08) | 0.06 | (0.04) | 0.50 | (0.07) | 0.04 | (0.02) | 0.53 | (0.07) | 0.06 | (0.04) |
|  |  | 2 | 0.53 | (0.08) | 0.06 | (0.04) | 0.51 | (0.09) | 0.05 | (0.04) | 0.53 | (0.08) | 0.06 | (0.04) |
|  |  | 4 | 0.54 | (0.09) | 0.07 | (0.05) | 0.52 | (0.08) | 0.07 | (0.05) | 0.54 | (0.09) | 0.07 | (0.04) |
| UGI | E | 1.5 | 0.61 | (0.05) | 0.25 | (0.23) | 0.52 | (0.03) | 0.18 | (0.11) | 0.61 | (0.08) | 0.30 | (0.25) |
|  |  | 2 | 0.61 | (0.06) | 0.25 | (0.23) | 0.52 | (0.02) | 0.18 | (0.14) | 0.61 | (0.08) | 0.29 | (0.24) |
|  |  | 4 | 0.58 | (0.08) | 0.24 | (0.23) | 0.56 | (0.02) | 0.16 | (0.12) | 0.59 | (0.11) | 0.27 | (0.22) |
|  | M | 1.5 | 0.62 | (0.03) | 0.26 | (0.22) | 0.57 | (0.04) | 0.22 | (0.15) | 0.61 | (0.05) | 0.29 | (0.25) |
|  |  | 2 | 0.61 | (0.04) | 0.25 | (0.24) | 0.56 | (0.05) | 0.19 | (0.16) | 0.61 | (0.06) | 0.29 | (0.26) |
|  |  | 4 | 0.56 | (0.08) | 0.21 | (0.21) | 0.56 | (0.05) | 0.15 | (0.12) | 0.56 | (0.09) | 0.25 | (0.23) |
|  | C | 1.5 | 0.61 | (0.10) | 0.26 | (0.22) | 0.51 | (0.04) | 0.17 | (0.10) | 0.61 | (0.10) | 0.26 | (0.22) |
|  |  | 2 | **0.64** | (0.09) | **0.26** | (0.21) | 0.54 | (0.03) | 0.17 | (0.13) | 0.64 | (0.09) | 0.26 | (0.21) |
|  |  | 4 | 0.62 | (0.09) | 0.24 | (0.20) | 0.56 | (0.03) | 0.16 | (0.14) | 0.62 | (0.09) | 0.24 | (0.20) |
