# Supplementary material for: Inhibitors of Helicobacter pylori Protease HtrA Found by ‘Virtual Ligand’ Screening Combat Bacterial Invasion of Epithelia
Source: PLoS One. 2011 Mar 31;6(3):e17986. doi: 10.1371/journal.pone.0017986 (PMC3069028; doi:10.1371/journal.pone.0017986)
Supplement: Table S3 — Results of retrospective screening using the COBRA database. Three different dissimilarity metrics were used a) Euclidian distance, b) Manhattan distance c) Cosine similarity. The highest ROC-AUC for each model is marked in bold. (DOCX) [file pone.0017986.s007.docx]

**Table S3.** Results of retrospective screening using the COBRA database. Three different dissimilarity metrics were used a) Euclidian distance, b) Manhattan distance c) Cosine similarity. The highest ROC-AUC for each model is marked in bold.

|  |  |  |  | no scaling | | | | block scaling | | | | scaling to one | | | |
| --- | --- | --- | --- | --- | --- | --- | --- | --- | --- | --- | --- | --- | --- | --- | --- |
| enzyme | PDB ID | pocket(s) ^1)^ | cluster radius [Å] ^2)^ | ROC-AUC | (σ) | BEDROC | (σ) | ROC-AUC | (σ) | BEDROC | (σ) | ROC-AUC | (σ) | BEDROC | (σ) |
| ACE^a)^ | 1o86 | 1 | 1.5 | 0.34 | (0.01) | 0.00 | (0.00) | **0.50** | (0.01) | 0.01 | (0.00) | 0.24 | (0.01) | 0.00 | (0.00) |
|  | 1o86 | 1 | 1.9 | 0.39 | (0.01) | 0.00 | (0.00) | 0.47 | (0.01) | 0.00 | (0.00) | 0.28 | (0.01) | 0.00 | (0.00) |
|  | 1o86 | 1 | 4 | 0.35 | (0.01) | 0.00 | (0.00) | 0.47 | (0.01) | 0.01 | (0.00) | 0.26 | (0.01) | 0.00 | (0.00) |
| ACE^b)^ | 1o86 | 1 | 1.5 | 0.32 | (0.01) | 0.00 | (0.00) | 0.40 | (0.01) | 0.00 | (0.00) | 0.26 | (0.01) | 0.00 | (0.00) |
|  | 1o86 | 1 | 1.9 | **0.42** | (0.01) | 0.00 | (0.00) | 0.42 | (0.01) | 0.00 | (0.00) | 0.35 | (0.01) | 0.00 | (0.00) |
|  | 1o86 | 1 | 4 | 0.33 | (0.01) | 0.00 | (0.00) | 0.40 | (0.02) | 0.01 | (0.00) | 0.28 | (0.01) | 0.00 | (0.00) |
| ACE^c)^ | 1o86 | 1 | 1.5 | 0.41 | (0.01) | 0.01 | (0.00) | 0.43 | (0.01) | 0.01 | (0.01) | 0.41 | (0.01) | 0.01 | (0.00) |
|  | 1o86 | 1 | 1.9 | **0.44** | (0.00) | 0.00 | (0.00) | 0.32 | (0.01) | 0.00 | (0.00) | 0.44 | (0.01) | 0.00 | (0.00) |
|  | 1o86 | 1 | 4 | 0.42 | (0.01) | 0.00 | (0.00) | 0.35 | (0.01) | 0.00 | (0.01) | 0.42 | (0.01) | 0.00 | (0.00) |
| COX-2^a)^ | 3pgh | 1 | 1.5 | 0.46 | (0.01) | 0.03 | (0.01) | 0.40 | (0.01) | 0.01 | (0.00) | **0.72** | (0.01) | 0.16 | (0.01) |
|  | 3pgh | 1 | 1.9 | 0.48 | (0.01) | 0.06 | (0.01) | 0.37 | (0.01) | 0.00 | (0.00) | 0.54 | (0.01) | 0.02 | (0.00) |
|  | 3pgh | 1 | 4 | 0.44 | (0.01) | 0.06 | (0.01) | 0.44 | (0.01) | 0.02 | (0.00) | 0.50 | (0.01) | 0.02 | (0.00) |
|  | 3pgh | 1 (part) ^3)^ | 1.5 | 0.53 | (0.02) | 0.13 | (0.01) | 0.37 | (0.01) | 0.04 | (0.01) | 0.75 | (0.01) | 0.28 | (0.02) |
|  | 3pgh | 1 (part) ^3)^ | 1.9 | 0.57 | (0.01) | 0.16 | (0.01) | 0.47 | (0.01) | 0.15 | (0.01) | 0.80 | (0.01) | 0.35 | (0.01) |
|  | 3pgh | 1 (part) ^3)^ | 4 | 0.55 | (0.01) | 0.19 | (0.01) | 0.60 | (0.02) | 0.27 | (0.02) | **0.81** | (0.01) | 0.36 | (0.01) |
|  | 5cox | 3 | 1.5 | 0.39 | (0.01) | 0.01 | (0.00) | 0.31 | (0.01) | 0.01 | (0.00) | 0.47 | (0.01) | 0.01 | (0.00) |
|  | 5cox | 3 | 1.9 | 0.41 | (0.01) | 0.02 | (0.00) | 0.37 | (0.01) | 0.00 | (0.00) | **0.50** | (0.01) | 0.01 | (0.00) |
|  | 5cox | 3 | 4 | 0.37 | (0.01) | 0.02 | (0.00) | 0.45 | (0.01) | 0.03 | (0.00) | 0.44 | (0.01) | 0.01 | (0.00) |
| COX-2^b)^ | 3pgh | 1 | 1.5 | 0.62 | (0.01) | 0.04 | (0.00) | 0.49 | (0.01) | 0.00 | (0.00) | 0.74 | (0.01) | 0.18 | (0.01) |
|  | 3pgh | 1 | 1.9 | 0.74 | (0.01) | 0.19 | (0.01) | 0.53 | (0.00) | 0.00 | (0.00) | 0.69 | (0.01) | 0.08 | (0.00) |
|  | 3pgh | 1 | 4 | **0.77** | (0.00) | 0.15 | (0.01) | 0.57 | (0.01) | 0.03 | (0.00) | 0.66 | (0.01) | 0.06 | (0.00) |
|  | 3pgh | 1 (part) ^3)^ | 1.5 | 0.69 | (0.01) | 0.11 | (0.01) | 0.42 | (0.01) | 0.04 | (0.01) | 0.76 | (0.01) | 0.21 | (0.01) |
|  | 3pgh | 1 (part) ^3)^ | 1.9 | 0.81 | (0.00) | 0.28 | (0.01) | 0.73 | (0.01) | 0.30 | (0.02) | 0.81 | (0.01) | 0.40 | (0.02) |
|  | 3pgh | 1 (part) ^3)^ | 4 | 0.84 | (0.01) | 0.29 | (0.01) | 0.82 | (0.00) | 0.36 | (0.01) | **0.85** | (0.00) | 0.44 | (0.01) |
|  | 5cox | 3 | 1.5 | 0.50 | (0.01) | 0.01 | (0.00) | 0.33 | (0.01) | 0.00 | (0.00) | 0.50 | (0.01) | 0.01 | (0.00) |
|  | 5cox | 3 | 1.9 | **0.63** | (0.00) | 0.06 | (0.00) | 0.53 | (0.01) | 0.02 | (0.00) | 0.62 | (0.01) | 0.04 | (0.00) |
|  | 5cox | 3 | 4 | 0.61 | (0.01) | 0.03 | (0.00) | 0.61 | (0.01) | 0.04 | (0.01) | 0.59 | (0.01) | 0.02 | (0.00) |
| COX-2^c)^ | 3pgh | 1 | 1.5 | 0.46 | (0.01) | 0.02 | (0.00) | **0.51** | (0.01) | 0.03 | (0.00) | 0.46 | (0.01) | 0.02 | (0.00) |
|  | 3pgh | 1 | 1.9 | 0.42 | (0.01) | 0.01 | (0.00) | 0.40 | (0.01) | 0.01 | (0.00) | 0.42 | (0.01) | 0.01 | (0.00) |
|  | 3pgh | 1 | 4 | 0.41 | (0.01) | 0.01 | (0.00) | 0.38 | (0.00) | 0.01 | (0.00) | 0.41 | (0.01) | 0.01 | (0.00) |
|  | 3pgh | 1 (part) ^3)^ | 1.5 | 0.48 | (0.01) | 0.21 | (0.00) | 0.37 | (0.01) | 0.05 | (0.00) | 0.49 | (0.01) | 0.21 | (0.01) |
|  | 3pgh | 1 (part) ^3)^ | 1.9 | 0.51 | (0.01) | 0.21 | (0.01) | 0.57 | (0.01) | 0.17 | (0.01) | 0.51 | (0.01) | 0.20 | (0.01) |
|  | 3pgh | 1 (part) ^3)^ | 4 | 0.52 | (0.01) | 0.21 | (0.01) | **0.68** | (0.01) | 0.27 | (0.01) | 0.51 | (0.01) | 0.21 | (0.00) |
|  | 5cox | 3 | 1.5 | 0.22 | (0.00) | 0.00 | (0.00) | 0.26 | (0.01) | 0.01 | (0.00) | 0.22 | (0.00) | 0.00 | (0.00) |
|  | 5cox | 3 | 1.9 | 0.25 | (0.01) | 0.00 | (0.00) | 0.44 | (0.01) | 0.01 | (0.00) | 0.25 | (0.00) | 0.00 | (0.00) |
|  | 5cox | 3 | 4 | 0.24 | (0.00) | 0.01 | (0.00) | **0.48** | (0.01) | 0.04 | (0.01) | 0.24 | (0.00) | 0.00 | (0.00) |
| DHFR^a)^ | 1kmv | 1 | 1.5 | 0.63 | (0.01) | 0.11 | (0.01) | 0.61 | (0.02) | 0.05 | (0.01) | 0.65 | (0.01) | 0.13 | (0.02) |
|  | 1kmv | 1 | 1.9 | 0.60 | (0.02) | 0.19 | (0.01) | 0.64 | (0.01) | 0.06 | (0.01) | 0.67 | (0.02) | 0.28 | (0.03) |
|  | 1kmv | 1 | 4 | 0.59 | (0.01) | 0.11 | (0.01) | **0.71** | (0.01) | 0.07 | (0.01) | 0.65 | (0.01) | 0.23 | (0.02) |
| DHFR^b)^ | 1kmv | 1 | 1.5 | 0.66 | (0.01) | 0.10 | (0.01) | 0.71 | (0.01) | 0.15 | (0.01) | 0.70 | (0.01) | 0.13 | (0.01) |
|  | 1kmv | 1 | 1.9 | 0.63 | (0.02) | 0.14 | (0.01) | 0.75 | (0.01) | 0.24 | (0.01) | 0.67 | (0.02) | 0.14 | (0.01) |
|  | 1kmv | 1 | 4 | 0.57 | (0.01) | 0.05 | (0.01) | **0.78** | (0.01) | 0.17 | (0.01) | 0.62 | (0.01) | 0.06 | (0.01) |
| DHFR^c)^ | 1kmv | 1 | 1.5 | 0.63 | (0.01) | 0.14 | (0.01) | 0.57 | (0.01) | 0.05 | (0.01) | 0.63 | (0.01) | 0.14 | (0.01) |
|  | 1kmv | 1 | 1.9 | 0.62 | (0.01) | 0.30 | (0.01) | 0.64 | (0.01) | 0.07 | (0.00) | 0.63 | (0.01) | 0.30 | (0.01) |
|  | 1kmv | 1 | 4 | 0.62 | (0.01) | 0.26 | (0.01) | **0.67** | (0.01) | 0.08 | (0.01) | 0.62 | (0.01) | 0.27 | (0.01) |
| fXa^a)^ | 2bok | 1 | 1.5 | **0.70** | (0.01) | 0.07 | (0.00) | 0.62 | (0.01) | 0.07 | (0.00) | 0.35 | (0.01) | 0.00 | (0.00) |
|  | 2bok | 1 | 1.9 | 0.64 | (0.01) | 0.04 | (0.00) | 0.69 | (0.01) | 0.12 | (0.01) | 0.32 | (0.01) | 0.00 | (0.00) |
|  | 2bok | 1 | 4 | 0.64 | (0.01) | 0.05 | (0.00) | 0.62 | (0.01) | 0.08 | (0.01) | 0.31 | (0.01) | 0.00 | (0.00) |
|  | 2bok | 1. 6. 13. 17 | 1.5 | 0.78 | (0.00) | 0.15 | (0.01) | 0.69 | (0.01) | 0.09 | (0.01) | **0.82** | (0.00) | 0.17 | (0.01) |
|  | 2bok | 1. 6. 13. 17 | 1.9 | 0.73 | (0.00) | 0.09 | (0.00) | 0.68 | (0.01) | 0.07 | (0.01) | 0.78 | (0.01) | 0.21 | (0.01) |
|  | 2bok | 1. 6. 13. 17 | 4 | 0.74 | (0.01) | 0.10 | (0.01) | 0.57 | (0.01) | 0.03 | (0.00) | 0.82 | (0.00) | 0.21 | (0.01) |
| fXa^b)^ | 2bok | 1 | 1.5 | 0.52 | (0.01) | 0.02 | (0.00) | 0.52 | (0.00) | 0.04 | (0.00) | 0.30 | (0.00) | 0.00 | (0.00) |
|  | 2bok | 1 | 1.9 | 0.41 | (0.01) | 0.00 | (0.00) | **0.64** | (0.01) | 0.05 | (0.00) | 0.29 | (0.00) | 0.00 | (0.00) |
|  | 2bok | 1 | 4 | 0.37 | (0.00) | 0.00 | (0.00) | 0.53 | (0.00) | 0.03 | (0.00) | 0.27 | (0.01) | 0.00 | (0.00) |
|  | 2bok | 1. 6. 13. 17 | 1.5 | 0.78 | (0.00) | 0.17 | (0.01) | 0.75 | (0.01) | 0.13 | (0.00) | **0.81** | (0.01) | 0.19 | (0.01) |
|  | 2bok | 1. 6. 13. 17 | 1.9 | 0.67 | (0.01) | 0.12 | (0.01) | 0.76 | (0.00) | 0.10 | (0.00) | 0.70 | (0.01) | 0.24 | (0.01) |
|  | 2bok | 1. 6. 13. 17 | 4 | 0.71 | (0.00) | 0.11 | (0.00) | 0.70 | (0.01) | 0.06 | (0.00) | 0.78 | (0.00) | 0.18 | (0.01) |
| fXa^c)^ | 2bok | 1 | 1.5 | 0.45 | (0.00) | 0.02 | (0.00) | 0.49 | (0.00) | 0.05 | (0.00) | 0.44 | (0.00) | 0.03 | (0.00) |
|  | 2bok | 1 | 1.9 | 0.65 | (0.00) | 0.06 | (0.00) | 0.59 | (0.01) | 0.03 | (0.00) | 0.65 | (0.00) | 0.06 | (0.00) |
|  | 2bok | 1 | 4 | 0.66 | (0.00) | 0.06 | (0.00) | 0.57 | (0.01) | 0.03 | (0.00) | **0.66** | (0.01) | 0.07 | (0.00) |
|  | 2bok | 1. 6. 13. 17 | 1.5 | 0.81 | (0.00) | 0.16 | (0.00) | 0.63 | (0.00) | 0.04 | (0.00) | 0.81 | (0.00) | 0.16 | (0.01) |
|  | 2bok | 1. 6. 13. 17 | 1.9 | 0.81 | (0.00) | 0.21 | (0.01) | 0.63 | (0.00) | 0.03 | (0.00) | 0.81 | (0.00) | 0.21 | (0.01) |
|  | 2bok | 1. 6. 13. 17 | 4 | **0.82** | (0.00) | 0.20 | (0.01) | 0.49 | (0.00) | 0.02 | (0.00) | 0.82 | (0.00) | 0.20 | (0.01) |
| PPARγ^a)^ | 1zgy | 1 | 1.5 | **0.57** | (0.02) | 0.06 | (0.01) | 0.53 | (0.02) | 0.03 | (0.01) | 0.54 | (0.02) | 0.06 | (0.01) |
|  | 1zgy | 1 | 1.9 | 0.56 | (0.02) | 0.04 | (0.01) | 0.55 | (0.02) | 0.06 | (0.01) | 0.53 | (0.02) | 0.07 | (0.01) |
|  | 1zgy | 1 | 4 | 0.52 | (0.02) | 0.04 | (0.01) | 0.54 | (0.01) | 0.03 | (0.01) | 0.51 | (0.02) | 0.07 | (0.01) |
| PPARγ^b)^ | 1zgy | 1 | 1.5 | 0.56 | (0.01) | 0.03 | (0.01) | 0.52 | (0.02) | 0.03 | (0.01) | 0.47 | (0.01) | 0.02 | (0.00) |
|  | 1zgy | 1 | 1.9 | **0.61** | (0.01) | 0.05 | (0.01) | 0.58 | (0.02) | 0.05 | (0.01) | 0.54 | (0.01) | 0.07 | (0.02) |
|  | 1zgy | 1 | 4 | 0.58 | (0.02) | 0.04 | (0.01) | 0.52 | (0.02) | 0.04 | (0.01) | 0.49 | (0.02) | 0.05 | (0.01) |
| PPARγ^c)^ | 1zgy | 1 | 1.5 | 0.53 | (0.01) | 0.05 | (0.00) | 0.46 | (0.01) | 0.03 | (0.00) | **0.54** | (0.01) | 0.05 | (0.01) |
|  | 1zgy | 1 | 1.9 | 0.53 | (0.01) | 0.05 | (0.01) | 0.51 | (0.01) | 0.03 | (0.00) | 0.53 | (0.01) | 0.06 | (0.01) |
|  | 1zgy | 1 | 4 | 0.51 | (0.02) | 0.06 | (0.02) | 0.44 | (0.02) | 0.02 | (0.00) | 0.51 | (0.01) | 0.06 | (0.01) |
| trypsin^a)^ | 1dpo | 1 | 1.5 | 0.61 | (0.02) | 0.05 | (0.02) | 0.65 | (0.02) | 0.05 | (0.01) | 0.46 | (0.04) | 0.01 | (0.00) |
|  | 1dpo | 1 | 1.9 | 0.65 | (0.03) | 0.08 | (0.01) | **0.67** | (0.02) | 0.05 | (0.01) | 0.53 | (0.03) | 0.02 | (0.00) |
|  | 1dpo | 1 | 4 | 0.61 | (0.04) | 0.05 | (0.01) | 0.60 | (0.02) | 0.02 | (0.00) | 0.49 | (0.02) | 0.01 | (0.00) |
| trypsin^B)^ | 1dpo | 1 | 1.5 | 0.44 | (0.01) | 0.01 | (0.00) | **0.55** | (0.01) | 0.03 | (0.01) | 0.34 | (0.02) | 0.00 | (0.00) |
|  | 1dpo | 1 | 1.9 | 0.48 | (0.02) | 0.00 | (0.00) | 0.53 | (0.02) | 0.01 | (0.00) | 0.39 | (0.02) | 0.00 | (0.00) |
|  | 1dpo | 1 | 4 | 0.32 | (0.02) | 0.00 | (0.00) | 0.47 | (0.01) | 0.00 | (0.00) | 0.29 | (0.02) | 0.00 | (0.00) |
| trypsin^c)^ | 1dpo | 1 | 1.5 | 0.68 | (0.01) | 0.04 | (0.01) | 0.65 | (0.01) | 0.04 | (0.01) | 0.68 | (0.01) | 0.04 | (0.01) |
|  | 1dpo | 1 | 1.9 | **0.70** | (0.02) | 0.05 | (0.01) | 0.67 | (0.01) | 0.04 | (0.01) | 0.69 | (0.01) | 0.05 | (0.01) |
|  | 1dpo | 1 | 4 | 0.68 | (0.01) | 0.05 | (0.01) | 0.64 | (0.02) | 0.02 | (0.00) | 0.68 | (0.01) | 0.04 | (0.01) |
| tryptase^a)^ | 2fpz | 2. 4. 17 | 1.5 | 0.71 | (0.03) | 0.17 | (0.04) | 0.65 | (0.03) | 0.09 | (0.03) | 0.74 | (0.02) | 0.15 | (0.02) |
|  | 2fpz | 2. 4. 17 | 1.9 | 0.72 | (0.02) | 0.22 | (0.03) | 0.71 | (0.02) | 0.26 | (0.05) | **0.74** | (0.03) | 0.18 | (0.02) |
|  | 2fpz | 2. 4. 17 | 4 | 0.68 | (0.03) | 0.21 | (0.05) | 0.70 | (0.03) | 0.13 | (0.03) | 0.67 | (0.03) | 0.13 | (0.02) |
| tryptase^b)^ | 2fpz | 1 | 1.5 | 0.72 | (0.03) | 0.16 | (0.03) | 0.73 | (0.01) | 0.13 | (0.03) | 0.72 | (0.01) | 0.16 | (0.04) |
|  | 2fpz | 1 | 1.9 | **0.73** | (0.04) | 0.20 | (0.03) | 0.72 | (0.02) | 0.23 | (0.03) | 0.70 | (0.02) | 0.10 | (0.02) |
|  | 2fpz | 1 | 4 | 0.60 | (0.03) | 0.14 | (0.02) | 0.71 | (0.01) | 0.10 | (0.02) | 0.56 | (0.03) | 0.07 | (0.02) |
| tryptase^c)^ | 2fpz | 1 | 1.5 | 0.78 | (0.01) | 0.21 | (0.02) | 0.65 | (0.01) | 0.09 | (0.01) | 0.79 | (0.02) | 0.20 | (0.04) |
|  | 2fpz | 1 | 1.9 | **0.81** | (0.02) | 0.28 | (0.04) | 0.71 | (0.01) | 0.25 | (0.02) | 0.80 | (0.01) | 0.28 | (0.02) |
|  | 2fpz | 1 | 4 | 0.78 | (0.01) | 0.24 | (0.02) | 0.72 | (0.02) | 0.13 | (0.02) | 0.78 | (0.02) | 0.24 | (0.04) |
| UPA^a)^ | 2o8t | 1 | 1.5 | 0.64 | (0.01) | 0.10 | (0.01) | 0.51 | (0.02) | 0.04 | (0.02) | 0.65 | (0.03) | 0.15 | (0.02) |
|  | 2o8t | 1 | 1.9 | 0.74 | (0.02) | 0.23 | (0.03) | 0.58 | (0.02) | 0.04 | (0.01) | **0.78** | (0.02) | 0.27 | (0.01) |
|  | 2o8t | 1 | 4 | 0.67 | (0.02) | 0.18 | (0.02) | 0.66 | (0.01) | 0.18 | (0.02) | 0.72 | (0.02) | 0.26 | (0.02) |
| UPA^b)^ | 2o8t | 2. 4. 17 | 1.5 | 0.66 | (0.02) | 0.12 | (0.02) | 0.58 | (0.02) | 0.09 | (0.01) | 0.64 | (0.03) | 0.16 | (0.02) |
|  | 2o8t | 2. 4. 17 | 1.9 | 0.75 | (0.02) | 0.20 | (0.02) | 0.67 | (0.02) | 0.10 | (0.01) | **0.75** | (0.02) | 0.25 | (0.02) |
|  | 2o8t | 2. 4. 17 | 4 | 0.66 | (0.03) | 0.28 | (0.03) | 0.73 | (0.02) | 0.22 | (0.02) | 0.66 | (0.02) | 0.29 | (0.02) |
| UPA^c)^ | 2o8t | 2. 4. 17 | 1.5 | 0.68 | (0.02) | 0.13 | (0.01) | 0.47 | (0.01) | 0.04 | (0.01) | 0.68 | (0.01) | 0.14 | (0.01) |
|  | 2o8t | 2. 4. 17 | 1.9 | 0.74 | (0.02) | 0.25 | (0.01) | 0.63 | (0.01) | 0.07 | (0.01) | **0.74** | (0.01) | 0.27 | (0.01) |
|  | 2o8t | 2. 4. 17 | 4 | 0.71 | (0.02) | 0.23 | (0.01) | 0.72 | (0.01) | 0.20 | (0.02) | 0.70 | (0.02) | 0.23 | (0.02) |

1) The numbering corresponds to the PocketPicker output.

2) LIQUID cluster radius for lipophilic interactions.

3) Only a part of the pocket was used for virtual ligand screening.
