# Supplementary material for: Inhibitors of Helicobacter pylori Protease HtrA Found by ‘Virtual Ligand’ Screening Combat Bacterial Invasion of Epithelia
Source: PLoS One. 2011 Mar 31;6(3):e17986. doi: 10.1371/journal.pone.0017986 (PMC3069028; doi:10.1371/journal.pone.0017986)
Supplement: Table S6 — Structures and activities (inhibition of HtrA) of the inhibitory compounds, ordered according to falling inhibitory activity. The Gold docking rank calculated for all 26 ordered compounds as well as the Gold ASP score in brackets is shown in column 5. (DOCX) [file pone.0017986.s010.docx]

**Table S6.** Structures and activities (inhibition of E-cadherin cleavage by HtrA) of the inhibitory compounds, ordered according to falling inhibitory activity. The Gold docking rank calculated for all 26 ordered compounds as well as the Gold ASP score in brackets is shown in column 5.

| No. | Structure | Measured activity  (% inhibition) | Found by model | Gold Rank (Gold Score) |
| --- | --- | --- | --- | --- |
| **1** | 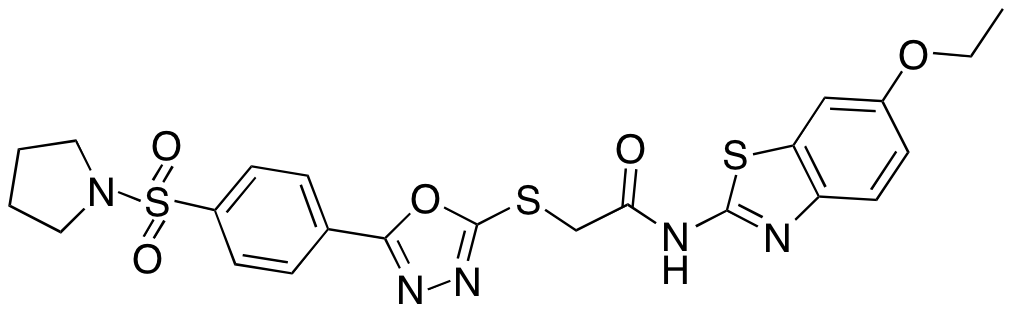 | 66 ± 28 | 1 | 5 (19) |
| **2** | 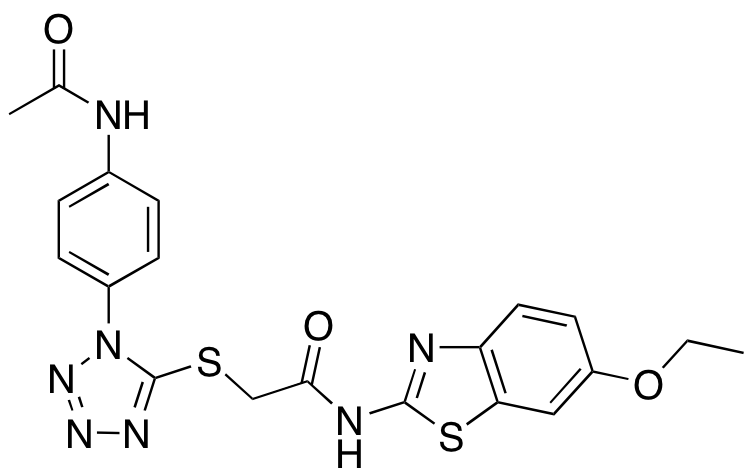 | 60 ± 53 | 1 | 1 (22) |
| **3** | 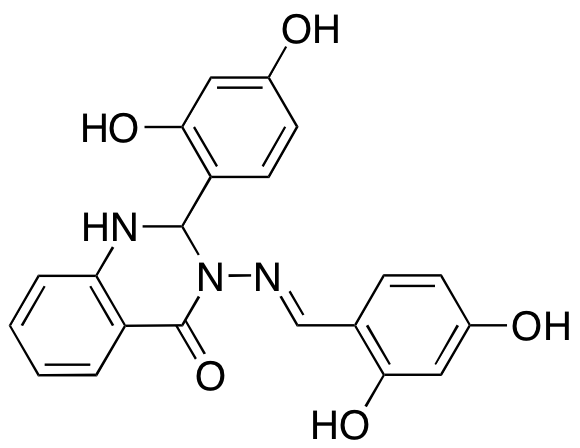 | 57 ± 58 | 3 | 23 (7) |
| **4** | 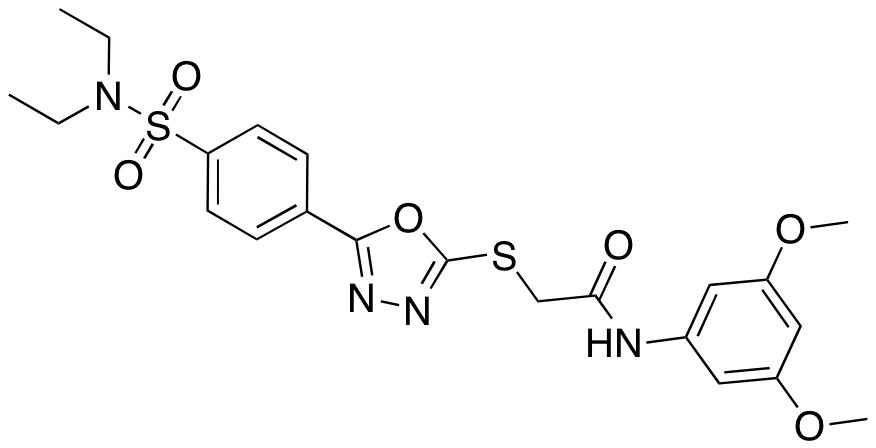 | 53 ± 33 | 1 | 16 (10) |
| **5** | 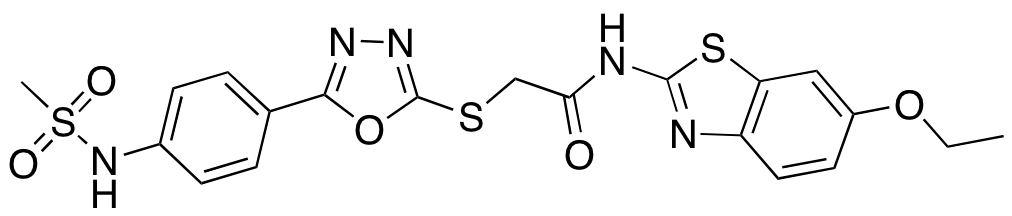 | 39 ± 31 | 1 | 8 (17) |
| **6** | 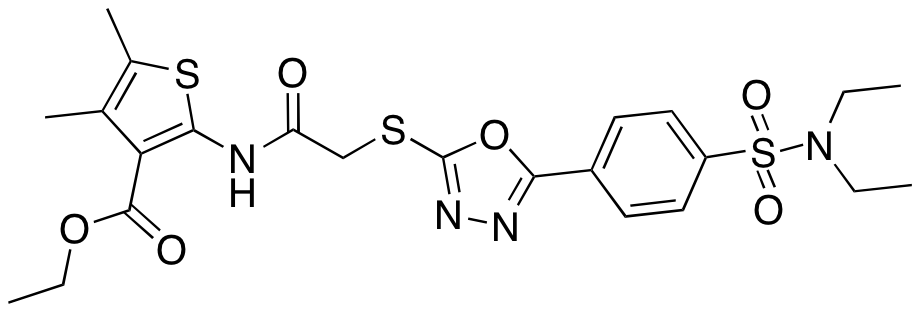 | 20 ± 9 | 1 | 10 (12) |
| **7** | 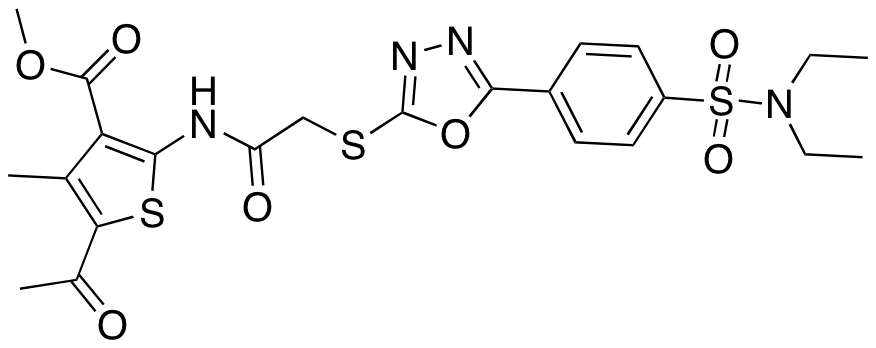 | inactive | 1 | 25 (3) |
| **8** | 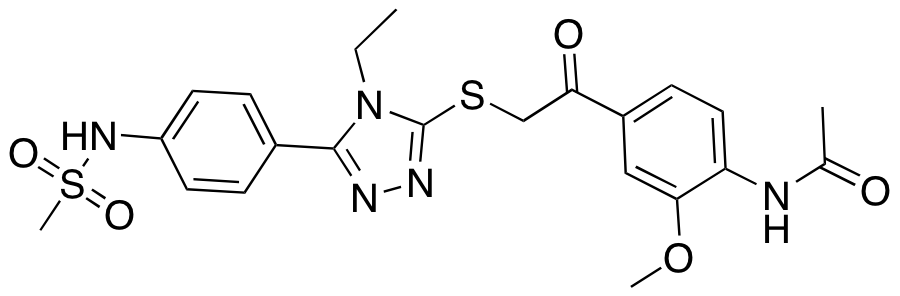 | inactive | 1 | 14 (11) |
| **9** | 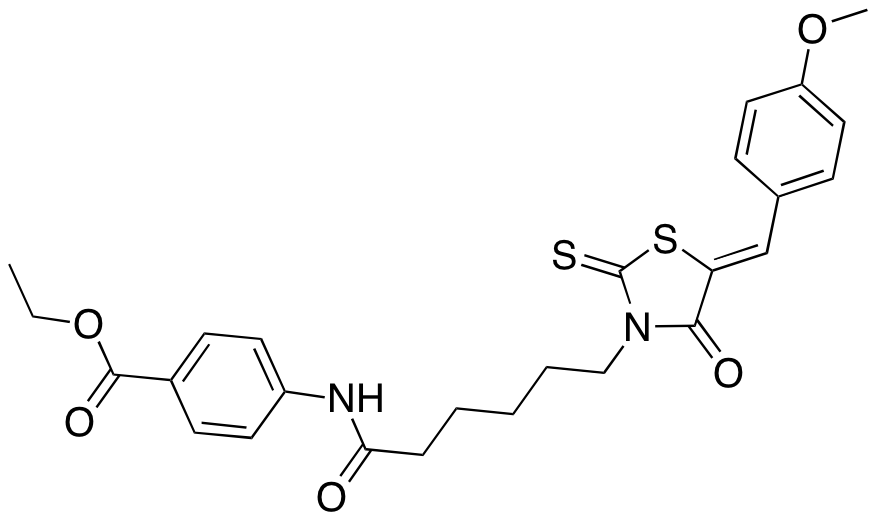 | inactive | 1 | 4 (19) |
| **10** | 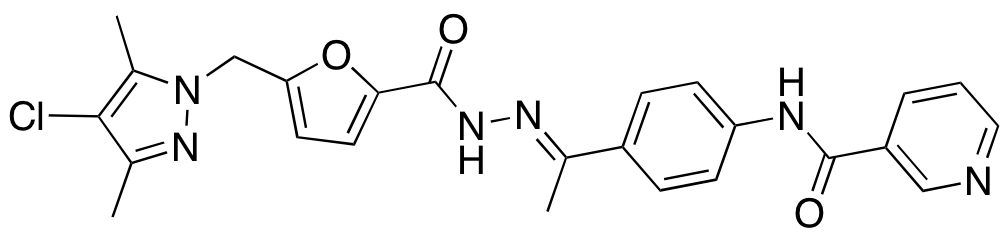 | inactive | 1 | 6 (18) |
| **11** | 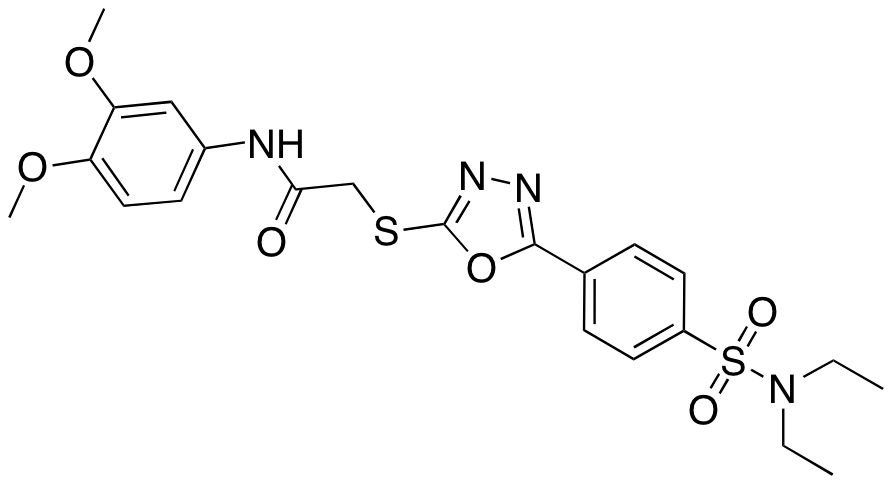 | inactive | 1 | 15 (10) |
| **12** | 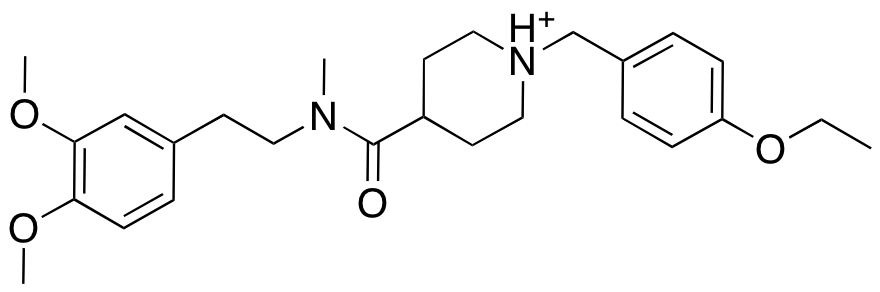 | inactive | 1 | 9 (15) |
| **13** | 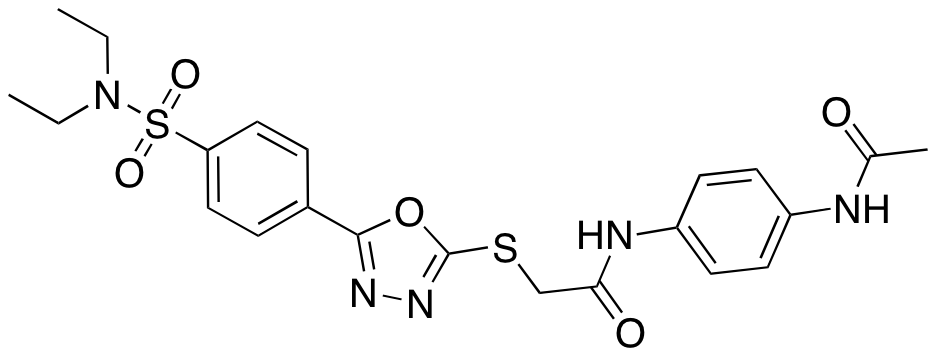 | inactive | 1 | 3 (19) |
| **14** | 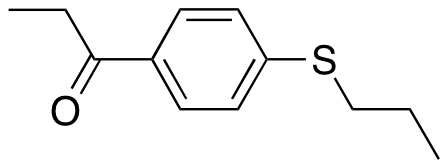 | inactive | 2 | 12 (11) |
| **15** | 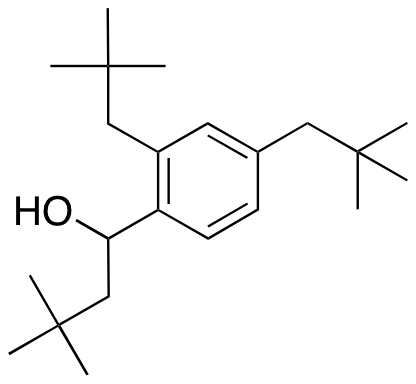 | inactive | 2 | 21 (7) |
| **16** | 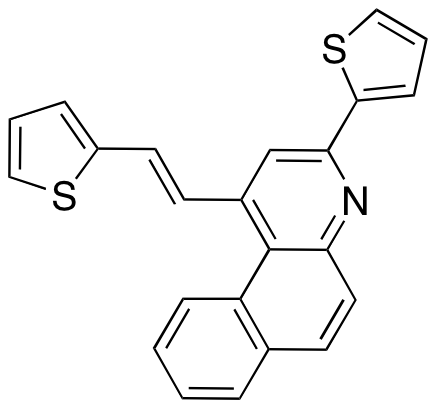 | inactive | 2 | 24 (7) |
| **17** | 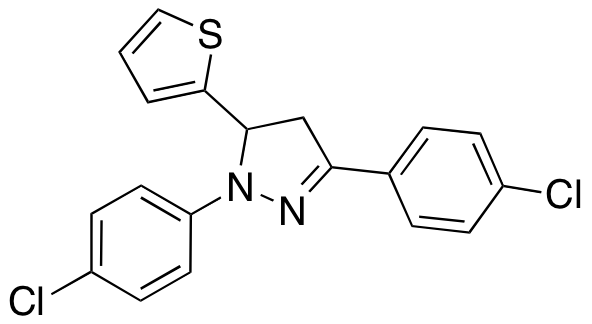 | inactive | 2 | 26 (0) |
| **18** | 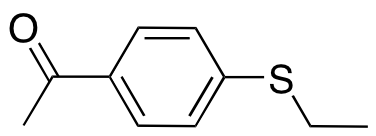 | inactive | 2 | 18 (9) |
| **19** | 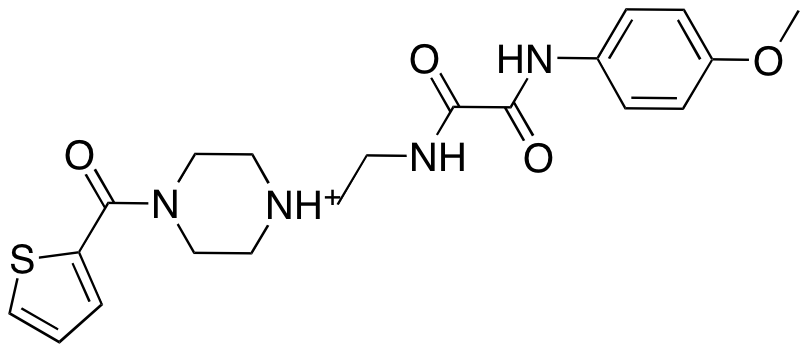 | inactive | 2 | 13 (11) |
| **20** | 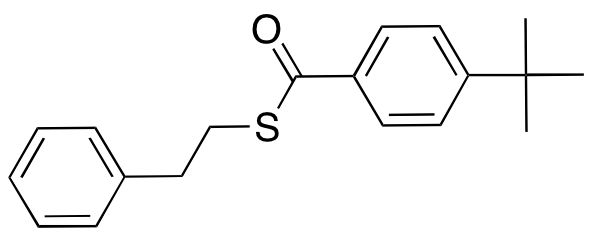 | inactive | 2 | 20 (8) |
| **21** | 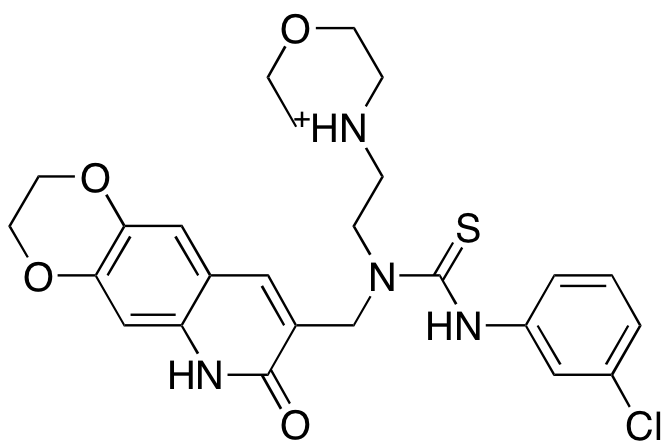 | potentially weakly active  (but considered as inactive) | 2 | 17 (10) |
| **22** | 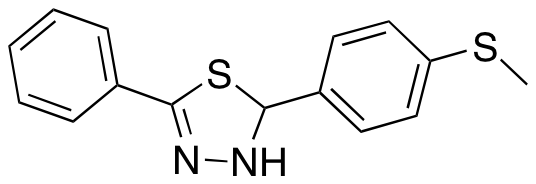 | inactive | 3 | 19 (9) |
| **23** | 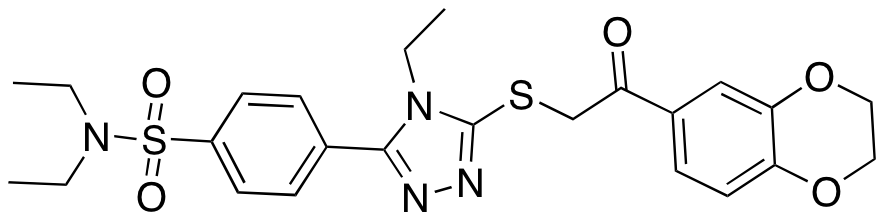 | insoluble | 1 | 22 (7) |
| **24** | 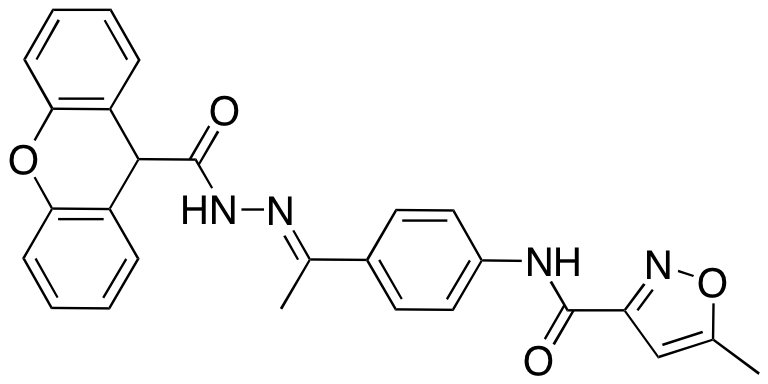 | insoluble | 1 | 2 (19) |
| **25** | 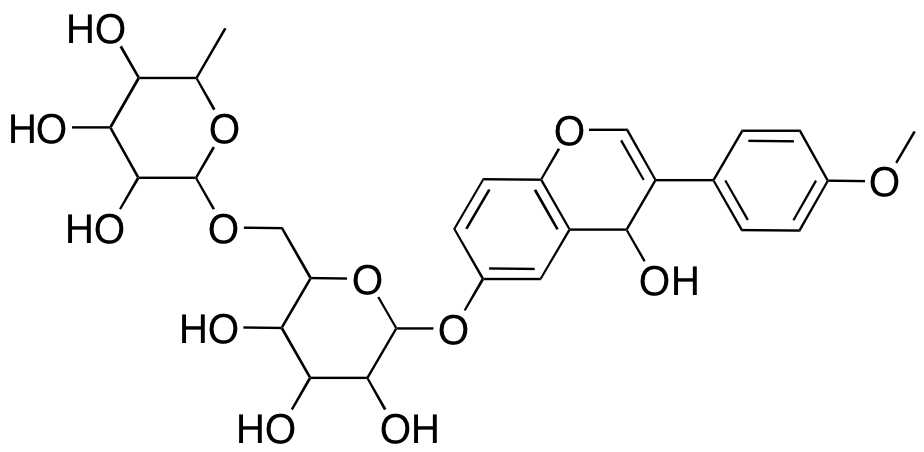 | insoluble | 2 | 7 (18) |
| **26** | 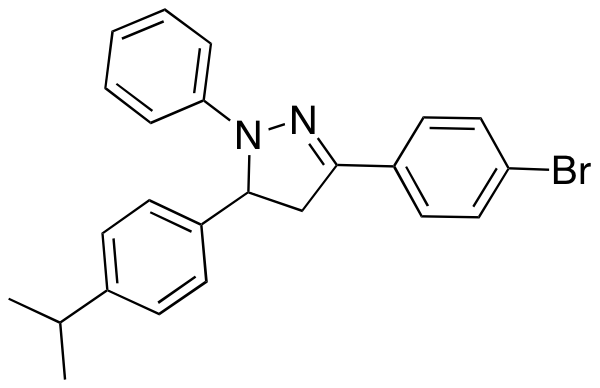 | insoluble | 2 | 11 (12) |
